# Osmotic signaling governs sunscreen biosynthesis to safeguard desert cyanobacteria against desiccation

**DOI:** 10.1002/mlf2.70075

**Published:** 2026-04-10

**Authors:** Lei Zhao, Hai‐Feng Xu, Jin‐Long Shang, Yong Li, Xiao‐Yue Yin, Zhong‐Chun Zhang, Huazhong Shi, Bao‐Sheng Qiu

**Affiliations:** ^1^ School of Life Sciences, Key Laboratory of Pesticide & Chemical Biology of Ministry of Education, Hubei Key Laboratory of Genetic Regulation and Integrative Biology, Central China Normal University Wuhan Hubei China; ^2^ Department of Chemistry and Biochemistry Texas Tech University Lubbock Texas USA

**Keywords:** cyanobacteria, desiccation tolerance, mycosporine‐like amino acids, osmoprotectant, sunscreen

## Abstract

Mycosporine‐like amino acids (MAAs) are natural sunscreens synthesized by a wide range of organisms. Although the induction of MAA production by ultraviolet radiation is well established, the signaling pathways involved and specific functions of MAAs under other stress conditions remain poorly understood. We demonstrated that MAAs serve as effective osmoprotectants for desiccation tolerance in the desert cyanobacterium *Nostoc flagelliforme*. Genetic disruption of genes encoding MAA biosynthetic enzymes eliminated MAA production, resulting in elevated oxidative damage, increased lipid peroxidation, and impaired photosynthesis under dehydration. Biochemical assays revealed that MAAs stabilize proteins and scavenge reactive oxygen species, indicating dual roles as osmolytes and antioxidants. Furthermore, we identified a signaling pathway Dsp1–OrrA that mediates the osmotic induction of MAA biosynthesis. Genetic disruption of either gene of Dsp1 and OrrA abolished osmotic induction and severely reduced desiccation tolerance. Phylogenomic analysis suggests that MAA biosynthesis is an ancient trait conserved in desiccation‐tolerant cyanobacteria. This work deepens our understanding of microbial adaptation to extreme environments and provides a foundation for synthetic biology applications of MAAs.

## Impact statement

Understanding how organisms survive desiccation is a fundamental biological problem. This study elucidates a crucial role for natural sunscreens (mycosporine‐like amino acids, MAAs) as vital protectants against desiccation in extremophile microbes. Our identification of the molecular pathway that senses water loss and activates MAA production uncovers a key survival mechanism for arid environments. Consequently, MAAs are redefined as multifunctional guardians, acting both as a sunscreen and as a desiccation shield, which establishes a novel paradigm for stress adaptation. This work provides the foundation for engineering microbial desiccation tolerance and enabling the sustainable production of these valuable compounds for biotechnological and agricultural applications.

## INTRODUCTION

Mycosporine‐like amino acids (MAAs) are small, water‐soluble secondary metabolites synthesized by diverse organisms, including cyanobacteria, algae, fungi, corals, and some invertebrates^1^, ^2^, ^3^, ^4^, ^5^, ^6^. Characterized by unique structural and functional properties, MAAs efficiently absorb ultraviolet radiation (UVR) and dissipate it as heat without generating free radical^7^, ^8^. This potent photoprotective capacity has driven both intensive research and also the development of commercial sunscreen applications. MAA biosynthesis has been linked to the shikimate pathway^9^, ^10^, ^11^ as well as an alternative route involving sedoheptulose‐7‐phosphate in the pentose phosphate pathway^12^. Intriguingly, vertebrates such as zebrafish also produce a related sunscreen compound, gadusol, highlighting the evolutionary conservation of this metabolite family^6^, ^13^. Given their vital roles in ecologically unique organisms, MAAs are considered as key molecules for environmental adaptation^14^.

Numerous studies have confirmed the photoprotective role of MAAs, with intense sunlight inducing their biosynthesis as an adaptive response to UVR stress^1^, ^13^, ^15^, ^16^. However, the photoprotective efficacy of intracellular MAAs is dependent on cell size^8^. Significant UVR shielding occurs in cells exceeding 10 μm in diameter, whereas smaller cells benefit modestly from cytoplasmic MAAs, suggesting that these compounds may play additional roles in microbial adaptation to other environmental stresses. This notion is supported by the observations that MAA biosynthesis is induced not only by UVR but also by salinity and desiccation in cyanobacteria^2^, ^17^. Nevertheless, MAAs typically accumulate at lower concentrations than canonical osmolytes, such as sucrose or glycine betaine, resulting in their classification as supplementary osmolytes^8^, ^18^, ^19^. Recent evidence demonstrates interspecific diversification of osmotic protectants and their role in enhancing desiccation tolerance^20^, ^21^. Notably, intracellular MAA concentrations reach ≥100 mM in cyanobacteria from hypersaline benthic crusts^2^, and comprise up to 3.2% dry weight in desiccated *Nostoc flagelliforme* colonies^22^. These exceptional accumulations raise a compelling question: Could MAAs serve as prominent osmolytes in microorganisms evolutionarily adapted to hypersaline or xeric extremes?

Desiccation tolerance is one of the most extraordinary survival strategies in nature, with elucidation of organismal survival mechanisms representing a fundamental challenge in biology^23^, ^24^, ^25^, ^26^. Osmoprotection is essential for maintaining cellular homeostasis during this process. Desert‐inhabiting cyanobacteria, featuring well‐characterized MAA biosynthesis pathways and simplified cellular structure, serve as unique model systems for elucidating the role of MAAs in osmoprotection^27^, ^28^, ^29^, ^30^. A five‐gene cluster, designated *mysABDC2C3* and involved in MAA biosynthesis, was identified in the desert cyanobacterium *N. flagelliforme*. The enzymes encoded in this cluster sequentially catalyze the formation of a novel MAA (M‐2‐DO), which features a linear structure composed of three 4‐deoxygadusol units connected by two ornithine residues^29^. Crucially, this species currently stands as the sole genetically tractable desert cyanobacterium amenable to targeted gene knockout^31^. Our previous work demonstrated that transcription factor OrrA binds to the *mysA* promoter in *N. flagelliforme* and that its overexpression enhances MAA production^28^. To date, OrrA is the only characterized prokaryotic regulator in MAA biosynthesis. However, significant knowledge gaps persist regarding the osmoprotective function of MAAs and the upstream signaling pathways governing osmotic induction of MAA biosynthesis.

In this study, we provided compelling evidence that MAAs function as effective osmoprotectants, with induced accumulation enhancing desiccation tolerance through protein stabilization and Reactive Oxygen Species (ROS) scavenging. We further identified the previously uncharacterized two‐component signaling system Dsp1–OrrA as a central molecular switch that specifically controls the osmotic induction of MAA biosynthesis, rather than the UVR‐induced production. These findings redefine the functional paradigm of MAAs by demonstrating their essential role as osmoprotectants in desiccation tolerance and reveal a novel regulatory axis underpinning stress adaptation in extremophilic microorganisms.

## RESULTS

### MAAs are required for desiccation tolerance

**Figure 1:**
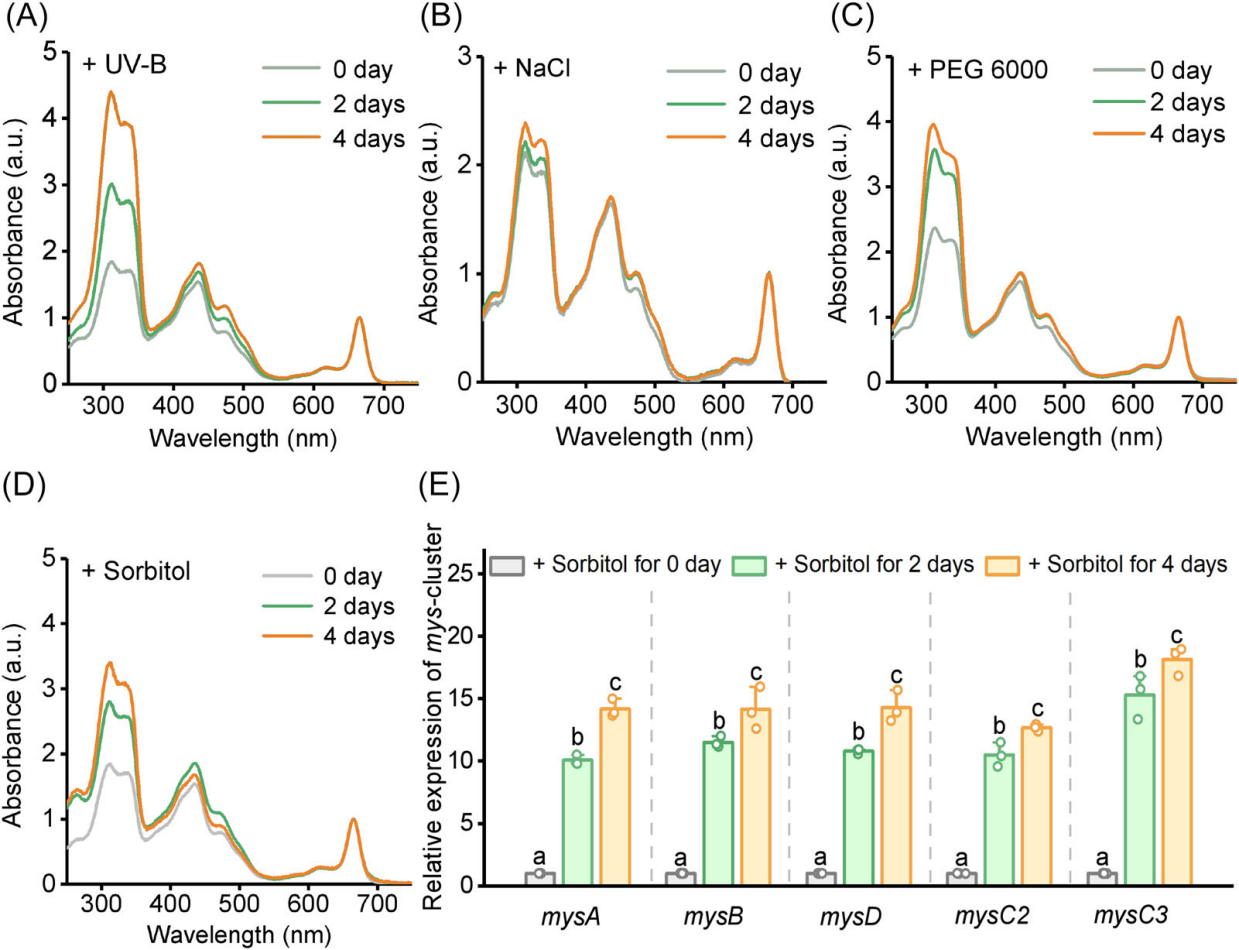
The biosynthesis of MAAs is significantly induced by water‐deficit stress. (A–D) Ultraviolet (UV) absorption spectra of MAAs (with a peak wavelength at 310 nm) in *Nostoc flagelliforme* after treatment for 0, 2, and 4 days with 0.2 W m^−2^ UV‐B (A), 0.05 M NaCl (B), 15% (wt/vol) polyethylene glycol (PEG) 6000 (C), or 0.3 M sorbitol (D), respectively. The absorption peaks of each sample were normalized to the chlorophyll *a* content. (E) Transcript levels of the *mys* cluster in *N. flagelliforme* treated with 0.3 M sorbitol for 0, 2, and 4 days. The values were normalized to 16S rRNA levels and presented relative to the transcript levels on Day 0. Data are shown as the mean ± SD of three independent replicates. Different lowercase letters above the error bars in (E) indicate significant differences (*p* < 0.05, Tukey's HSD). MAA, mycosporine‐like amino acids.

To elucidate the function of MAAs in *N. flagelliforme*, we selected three representative conditions mimicking the abiotic stresses that the cyanobacterium encounters in nature and assessed the response of MAA biosynthesis. MAAs are well known to be induced by UV‐B exposure^28^, ^32^, ^33^. Consistently, we observed markedly increased MAA accumulation when treated with 0.2 W m^−2^ UV‐B (Figure 1A) and a modest increase under 0.05 M NaCl treatment (Figure 1B), with MAA accounting for approximately 4% and 1% of cellular dry weight, respectively. For dehydration stress, we treated *N. flagelliforme* with high concentrations of polyethylene glycol (PEG) 6000 or sorbitol, which has been commonly used to mimic low water potential environments^31^, ^34^. Notably, supplementation with 15% PEG 6000 or 0.3 M sorbitol enhanced MAA production (Figure 1C, D), and after 4 days treatment with 0.3 M sorbitol, MAAs accounted for approximately 2% of cellular dry weight. Furthermore, both treatments upregulated the transcript levels of the MAA biosynthetic cluster genes (*mys* cluster). However, PEG 6000 only induced a marked upregulation on Day 2 (Figure S1), whereas sorbitol treatment resulted in sustained upregulation on both Day 2 and Day 4 (Figure 1E). These results indicate that in *N. flagelliforme*, MAA biosynthesis is not only induced by UV‐B but also responsive to dehydration, supporting a potential role of MAAs in desiccation tolerance.

**Figure 2:**
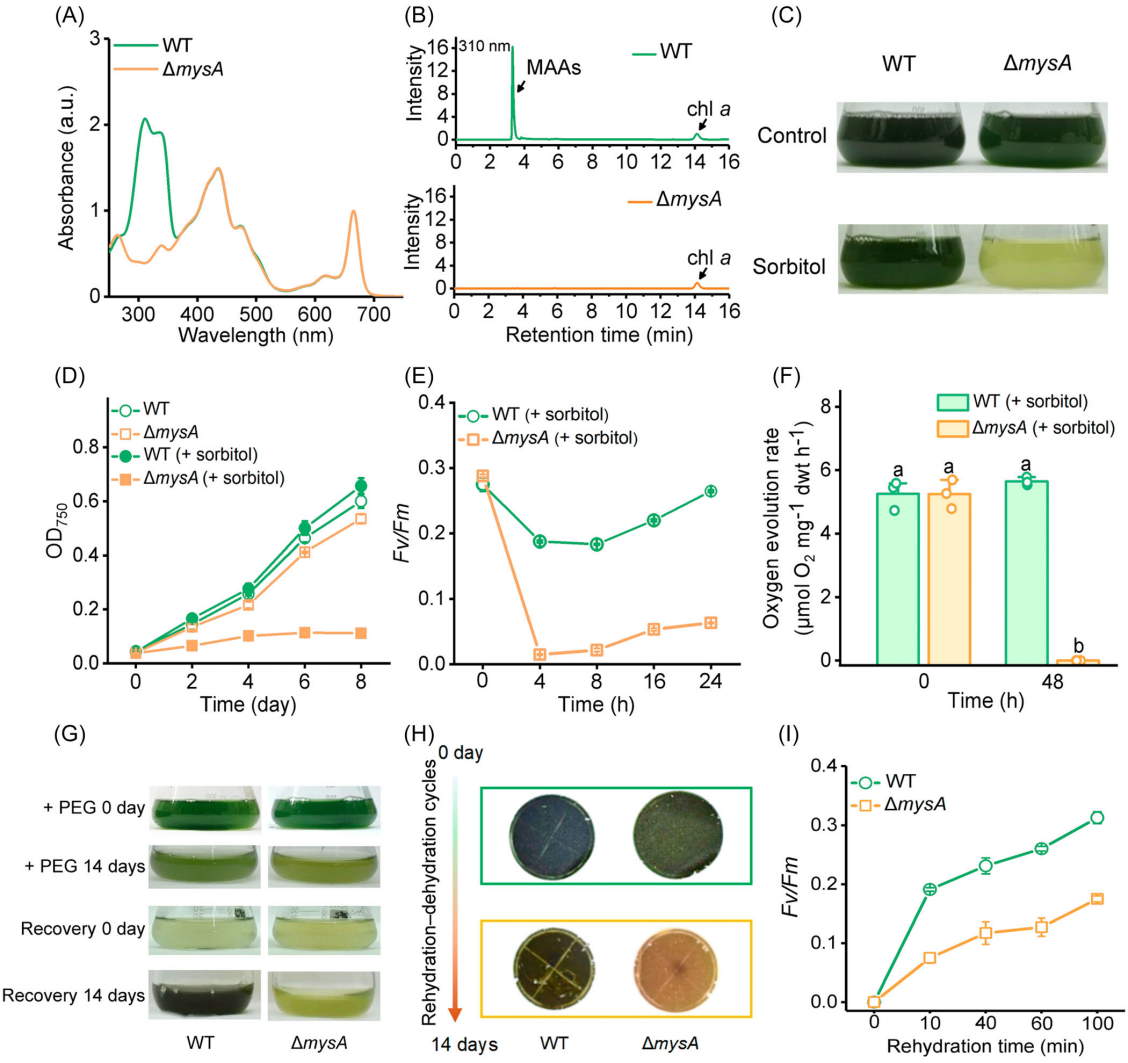
Deletion of *mysA* abolishes MAA biosynthesis and compromises water‐deficit stress tolerance in *N. flagelliforme*. (A, B) UV absorption spectra (A) and HPLC chromatograms (B) of MAAs in the wild‐type (WT) and Δ*mysA* strains. Absorption peaks were normalized to chlorophyll *a* content. Methanol extracts were analyzed by HPLC with detection at 310 nm. (C, D) Growth comparison of the WT and Δ*mysA* strains in BG11 medium. Cells were inoculated at an initial OD_750_ of 0.04 and grown under control conditions or with 0.3 M sorbitol to induce water‐deficit stress. Photographs were taken on Day 8 (C), and growth was monitored every 2 days (D). (E, F) Effects of short‐term water‐deficit stress (simulated by 0.3 M sorbitol) on *Fv/Fm* (E) and oxygen evolution rate (F) in the WT and Δ*mysA* strains. (G–I) Desiccation tolerance comparison between the WT and Δ*mysA* strains of *N. flagelliforme*. After 14 days of exposure to 22% (wt/vol) PEG 6000, the cultures were resuspended in fresh BG11 medium at an OD_750_ of 0.04 and incubated for an additional 14 days for recovery (G). Photographs (H) and *Fv/Fm* recovery (I) were taken after two weeks of rehydration–dehydration cycles. Data are shown as the mean ± SD of three independent replicates. Different lowercase letters above the error bars in (F) indicate significant differences between the WT and Δ*mysA* strains (*p* < 0.05, Student's *t*‐test). HPLC, high‐performance liquid chromatography.

We further used loss‐of‐function analysis to investigate the role of MAAs in *N. flagelliforme*. The first gene in the *mys* cluster, *mysA* (*COO91_06028*) encoding desmethyl‐4‐deoxygadusol synthase, was deleted using CRISPR‐Cpf1 gene‐editing technology, generating a *mysA* knockout mutant (Δ*mysA*). Analysis by ultraviolet (UV) absorbance spectrum and high‐performance liquid chromatography (HPLC) demonstrated that, when grown in standard BG11 medium, the Δ*mysA* strain completely lost the ability to synthesize MAAs compared to the wild‐type (WT) strain (Figure 2A,B). To assess the contribution of *mysA* to desiccation tolerance, we induced dehydration stress by treating *N. flagelliforme* with 0.3 M sorbitol. No phenotypic differences were observed between the mutant and WT strains under normal growth conditions, while Δ*mysA* growth was markedly inhibited by sorbitol treatment (Figure 2C,D). During the 8‐day culture period, the mutant showed reduced biomass accumulation and progressive bleaching when compared with the WT strain, indicating increased sensitivity to sorbitol‐induced dehydration stress.

Photosystem II (PSII) activity serves as a sensitive indicator of cell viability due to its high sensitivity to cellular water loss^35^. The photosynthetic performance of WT and Δ*mysA* strains under dehydration stress was assessed by measuring the maximal quantum efficiency of PSII (*Fv/Fm*). The results showed that, after 4 h of sorbitol treatment, the Δ*mysA* mutant showed a greater decrease in *Fv/Fm* values (0.01 ± 0.01) than the WT strain (0.18 ± 0.01) (Figure 2E). After treatment for 24 h, the WT strain retained 96.2% of its initial *Fv/Fm*, whereas Δ*mysA* retained only 21.9%. Furthermore, after 48 h of sorbitol treatment, the oxygen‐evolution rate in Δ*mysA* became nearly undetectable, which was significantly lower than that in the WT (Figure 2F). These results collectively indicate that the loss of *mysA* considerably impairs growth and photosynthetic activity under sorbitol‐induced dehydration stress, compromising *N. flagelliforme*'s tolerance to water loss.

To better mimic natural conditions, we then subjected the strains to prolonged PEG treatment and periodic dehydration–rehydration cycles. Under both treatments, the Δ*mysA* mutant showed markedly reduced water‐deficit stress tolerance compared to the WT (Figure 2G–I), thus providing direct evidence for the critical role of MAAs in desiccation tolerance. Moreover, phylogenetic analysis indicated that the gene cluster associated with MAA biosynthesis is selectively conserved during the evolution of terrestrial cyanobacteria (Figures S2 and S3), suggesting its important role in adaptation to arid environments.

The deletion of *mysA* not only abolishes MAA production but also prevents the synthesis of MAA precursor, 4‐deoxygadusol (4‐DG), which was reported to have antioxidant activity^16^. To determine whether the reduced desiccation tolerance in Δ*mysA* was primarily caused by MAA deficiency rather than the loss of 4‐DG, we generated a *mysC3* knockout mutant (Δ*mysC3*). HPLC analysis of methanol extracts at 310 nm revealed a distinct MAA peak at 2.7 min in the WT strain, which was not detected in the Δ*mysC3* strain (Figure S4A). When detected at 294 nm, a peak at 3.3 min in the Δ*mysC3* extract showed a UV‐absorption spectrum characteristic of 4‐DG. This peak was not observed in either the WT or the Δ*mysA* mutant (Figure S4B). This result indicats that Δ*mysC3* lacks MAAs but accumulates 4‐DG. Subsequently, we assessed the desiccation tolerance of Δ*mysC3* by analyzing the growth rate and photosynthetic activity. Under sorbitol treatment, Δ*mysC3* displayed slower growth rates and lower PSII activity than the WT strain (Figure S4C–E). Moreover, after 2 days of sorbitol treatment, Δ*mysC3* showed a substantially higher relative electrical conductivity (REC) (21.6% ± 3.4%) compared to the WT (15.3% ± 3.6%), indicating more severe plasma membrane damage (Figure S4F). Although the 4‐DG present in Δ*mysC3* appears to partially rescue the desiccation‐tolerant phenotype relative to Δ*mysA*, it remains insufficient to compensate for the effects resulting from the complete absence of MAAs in Δ*mysA*. Collectively, these results confirmed the indispensable role of MAAs in conferring tolerance to water deficit in *N. flagelliforme*.

### MAAs function as osmolytes and antioxidants in desiccation tolerance

**Figure 3:**
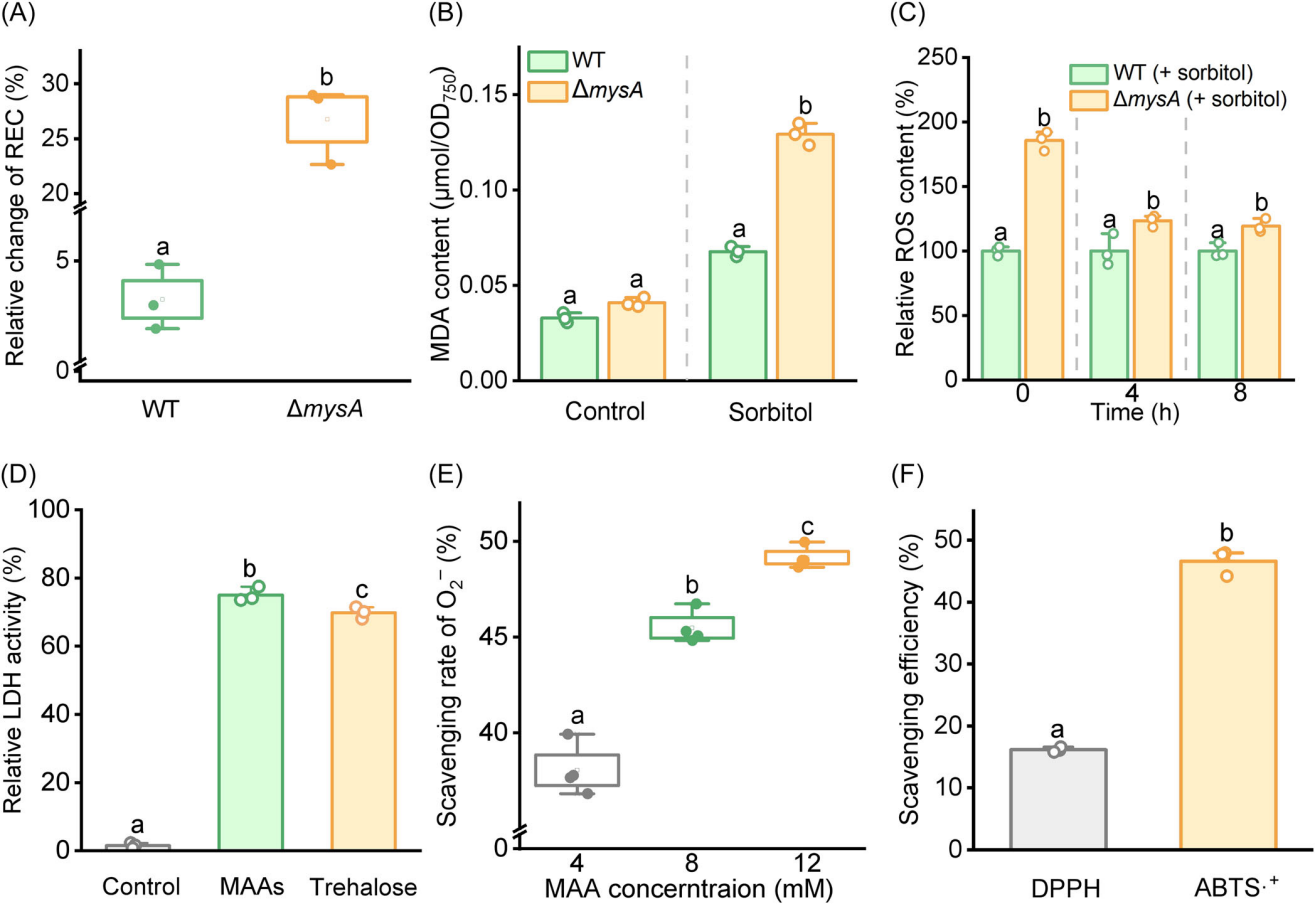
MAAs protect proteins against water deficit and scavenge reactive oxygen species (ROS). (A) Changes in the relative electrical conductivity (REC) in the WT and Δ*mysA* following treatment with 0.3 M sorbitol for 2 days (normalized to samples grown in BG11 medium). (B) Malondialdehyde (MDA) content in both the WT and Δ*mysA* after treatment with 0.3 M sorbitol for 2 days. MDA levels were normalized to OD_750_. (C) Relative levels of ROS in Δ*mysA* after exposure to 0.3 M sorbitol for 0, 4, and 8 h, normalized to the WT. (D) Enzyme activities of lactate dehydrogenase (LDH) subjected to dehydration in the absence (control) or presence of 30 mM MAAs. An equimolar trehalose solution served as a positive control. Following rehydration, enzyme activity was measured and normalized to that of the untreated control. (E) A dose‐dependent inhibition of superoxide anion radicals (O₂⁻) by MAAs observed at concentrations of 4, 8, and 12 mM. (F) Radical‐scavenging efficiency of 2.6 mM MAAs against 2,2‐Diphenyl‐1‐picrylhydrazyl (DPPH) and 2,2'‐azino‐bis(3 ‐ethylbenzothiazoline‐6‐sulfonic acid) (ABTS) radicals. Data are shown as the mean ± SD of three independent replicates. Different lowercase letters above the error bars indicate significant differences: for panels A, B, D, and F (*p* < 0.05, Student's *t*‐test), and for panels C and E (*p* < 0.05, Tukey's HSD).

Membrane integrity and fluidity are crucial for cyanobacteria survival in arid and desert environments^36^. To further elucidate the role of MAAs in desiccation tolerance of *N. flagelliforme*, we evaluated the physiological response of the strains to water deficit conditions. Following 2 days of sorbitol treatment, the Δ*mysA* mutant showed a significantly larger increase in REC (26.77% ± 3.56%) than the WT strain (4.23% ± 0.63%) (Figure 3A), indicating more severe membrane damage in the mutant. Under normal growth conditions, lipid peroxidation levels assessed by malondialdehyde (MDA) content showed no significant difference between WT and Δ*mysA* mutant. However, after treatment with 0.3 M sorbitol, the Δ*mysA* strain showed an MDA content 0.90 ± 0.01‐fold higher than that of the wild type (Figure 3B), which indicates elevated lipid peroxidation and oxidative stress in the MAA‐deficient mutant. Interestingly, the difference in ROS levels between the mutant and the wild type was more pronounced at 0 h than after sorbitol treatment (Figure 3C). This may be attributed to the reduced photosynthetic electron transport efficiency in the Δ*mysA* mutant, which could lead to lower photosynthetic electron leakage and consequently reduced ROS production under stress conditions. Together, these findings demonstrate that MAA deficiency increases oxidative damage during sorbitol‐induced dehydration in *N. flagelliforme*.

To further investigate the protective mechanisms of MAAs against desiccation, MAAs from *N. flagelliforme* were purified via HPLC fractionation, and the absorption spectrum of pure MAAs is shown in Figure S5A. Using *in vitro* assays, we evaluated the dehydration protection and antioxidant capacity of MAAs. As a well‐established biomarker for protectant efficacy, lactate dehydrogenase (LDH) undergoes irreversible deactivation upon dehydration due to its extreme desiccation sensitivity^24^. As shown in Figure 3D, complete dehydration and rehydration in the absence of protectants led to a total loss of LDH activity. In contrast, the presence of 30 mM of either MAAs or the known osmolyte trehalose conferred substantial protection during dehydration, with activity retention rates of 75.06% and 69.85%, respectively. This result indicates that MAAs can directly stabilize dehydration‐sensitive proteins during dehydration, similar to the established compatible solutes.

Water loss triggers significant ROS production in desiccation‐tolerant cyanobacteria, adversely impacting their survival in arid environments^37^. Consequently, activation of the antioxidant defense system is essential for the survival of desert cyanobacteria. While some MAAs have been reported to possess potential antioxidant properties^38^, the ROS‐scavenging capacity of the purified novel MAAs synthesized by *N. flagelliforme* remains poorly characterized. In our assays, the Δ*mysA* mutant showed higher ROS accumulation than the WT under both normal and stress conditions (Figures 3C and S5B). This is consistent with its more severe lipid peroxidation and oxidative stress (Figure 3A,B). We hypothesized that the excessive ROS accumulation observed in the Δ*mysA* mutant might be due to the absence of MAAs (Figure 3C). To test this, we conducted *in vitro* assays evaluating the ROS‐scavenging ability of MAAs isolated from *N. flagelliforme*. MAAs showed scavenging capability to superoxide anions, hydroxyl radicals, and singlet oxygen, with particularly higher efficiency for superoxide anions (Figures 3E and S5C). At 12 mM, MAAs scavenged 49.15% of superoxide anions (Figure 3E). They also showed potent activity against the water‐soluble 2,2‐Diphenyl‐1‐picrylhydrazyl (DPPH) and 2,2'‐azino‐bis(3 ‐ethylbenzothiazoline‐6‐sulfonic acid) (ABTS) radicals (Figure 3F). Collectively, these results establish that MAAs function as antioxidants, which contributes to desiccation tolerance in *N. flagelliforme* by mitigating oxidative stress during dehydration.

### Deletion of OrrA impairs osmotic induction of MAAs and reduces water‐deficit tolerance

**Figure 4:**
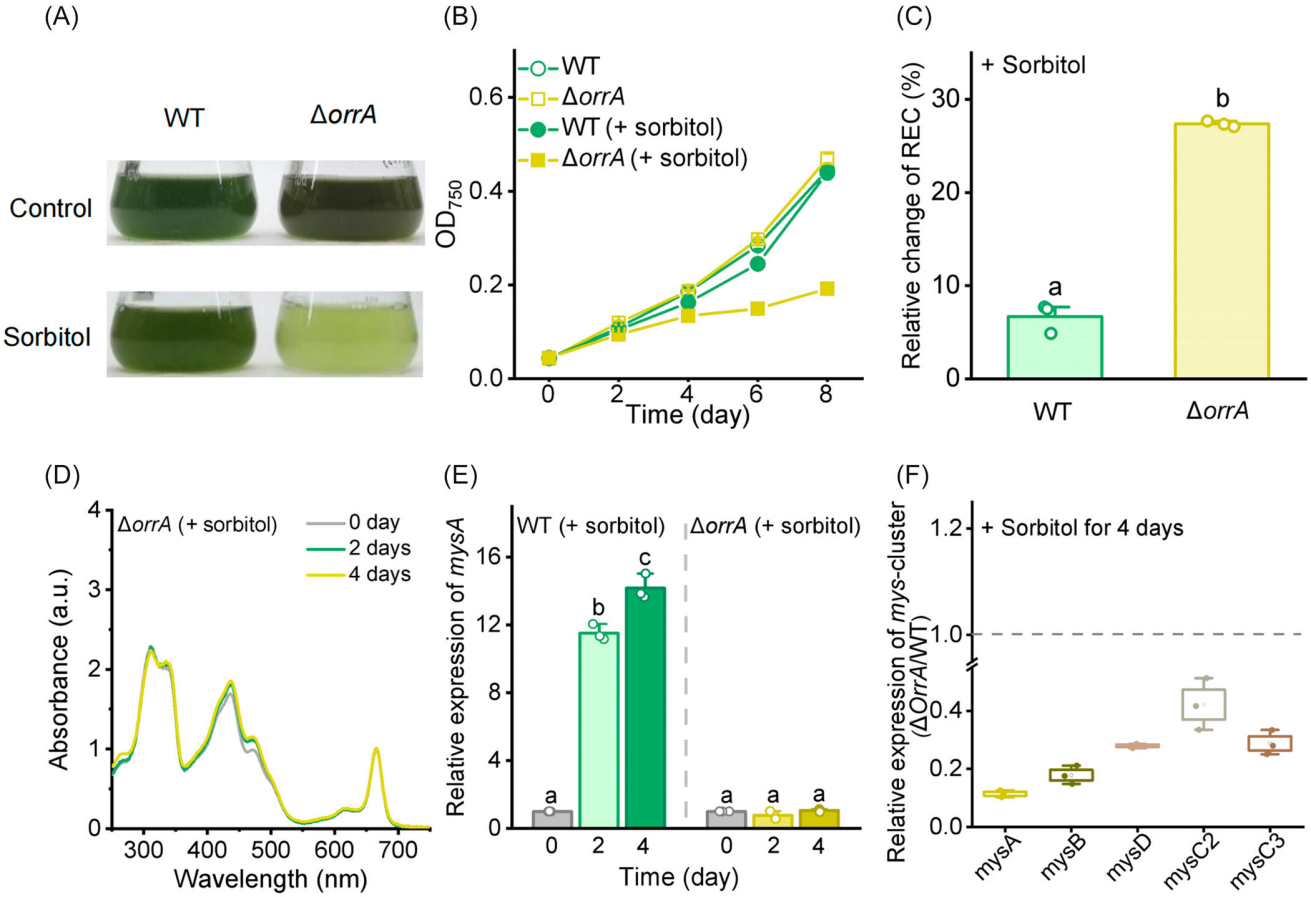
Deletion of OrrA impairs osmotic stress‐induced synthesis of MAAs and reduces water‐deficit tolerance in *N. flagelliforme*. (A, B) Growth comparison of WT and Δ*orrA* in BG11 medium. Cells were inoculated at an initial OD_750_ of 0.04 and grown under control conditions or with 0.3 M sorbitol to induce water‐deficit stress. Photographs were taken on Day 8 (A), and growth was monitored every 2 days (B). (C) Changes in REC levels in the WT and Δ*orrA* after 2 days of exposure to 0.3 M sorbitol, normalized to their respective untreated controls. (D) UV absorption spectra of MAAs in Δ*orrA* after 4 days of exposure to 0.3 M sorbitol. Absorption peaks were normalized to chlorophyll *a* content. (E) Relative transcript levels of *mysA* in the WT and Δ*orrA* under water‐deficit conditions for 4 days. Data were normalized to 16S rRNA and are presented as fold changes relative to the baseline level on Day 0. (F) Relative expression levels of the *mys* cluster in Δ*orrA* relative to the WT after 4 days of exposure to 0.3 M sorbitol. Data are shown as the mean ± SD of three independent replicates. Different letters above the error bars indicate significant differences: for panel (C) (*p* < 0.05, Student's *t*‐test) and for panel (E) (*p* < 0.05, Tukey's HSD).

To elucidate the signaling pathway underlying dehydration‐induced MAA biosynthesis, we investigated the transcriptional regulation of the *mys* cluster. Our previous study demonstrated that OrrA, a LuxR family transcription factor, can physically bind to the *mysA* promoter and regulate MAA biosynthesis under UV‐B radiation in *N. flagelliforme* ^28^. Interestingly, overexpression of OrrA accelerated the recovery of photosynthesis in *N. flagelliforme* during rehydration after dehydration^28^. However, the role of OrrA in regulating MAA biosynthesis under dehydration remains unclear. To test whether the dehydration‐tolerant phenotype involves enhanced MAA biosynthesis, we generated an *orrA* (*COO91_03246*) knockout mutant (Δ*orrA*) and compared its response to sorbitol‐induced dehydration with the WT strain. In liquid medium, addition of 0.3 M sorbitol markedly reduced the growth rate of Δ*orrA* but had minimal effect on the WT strain (Figure 4A,B). The Δ*orrA* mutant also showed substantially elevated REC (27.37% ± 0.30%) compared with the WT (6.70% ± 1.60%) (Figure 4C), indicating severe membrane damage of the mutant under dehydration. Notably, despite continuous 0.3 M sorbitol treatment for 4 days, MAA accumulation in Δ*orrA* remained unchanged (Figure 4D), and the transcript level of *mysA* in Δ*orrA* failed to increase relative to Day 0 (Figure 4E). Consistently, sorbitol‐treated Δ*orrA* showed lower transcript levels across the *mys* cluster compared to the WT strain (Figure 4F). These results establish that OrrA regulates MAA synthesis and thereby confers desiccation tolerance in *N. flagelliforme*. Strikingly, *orrA* transcription itself was unaffected by sorbitol treatment (Figure S6A), suggesting that upstream signaling components, rather than OrrA expression changes, mediate dehydration sensing.

### Dsp1 is an upstream dehydration‐responsive regulator of OrrA

**Figure 5:**
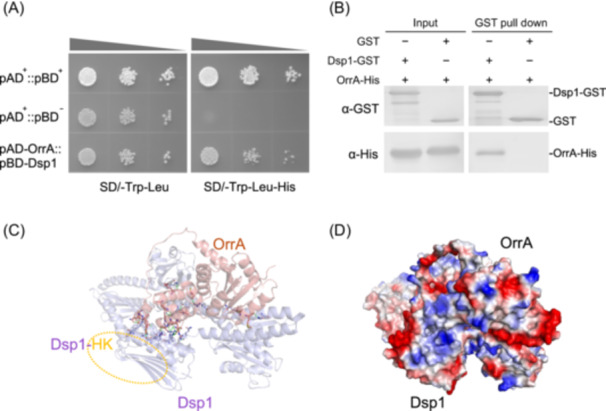
*In vitro* interaction analysis between Dsp1 and OrrA. (A) Interaction between Dsp1 and OrrA verified by a yeast two‐hybrid assay. Serial dilutions (OD_600_ = 0.1, 0.01, and 0.001; 10 µl each) of yeast transformants were spotted onto SD/‐Trp/‐Leu and SD/‐Trp/‐Leu/‐His agar plates and incubated for 3 days. (B) *In vitro* pull‐down assay of the interaction between Dsp1 and OrrA. GST was used as a negative control. (C, D) Interaction model of Dsp1 bound to OrrA. The structural models of Dsp1 and OrrA were predicted individually using AlphaFold3 (https://alphafoldserver.com/), and their docking interaction was analyzed with ClusPro (https://cluspro.bu.edu/login.php). The position enclosed by the orange dashed circle indicates the Dsp1‐HK domain, which is the main interaction site with OrrA (C). Electrostatic surface model of the Dsp1–OrrA complex, with electrostatic potentials calculated and visualized using PyMOL (Schrödinger, LLC) (D). HK, histidine kinase with ATPase.

To identify upstream regulators of OrrA, we used co‐immunoprecipitation (Co‐IP) to capture its interacting proteins. Liquid chromatography‐mass spectrometry (LC‐MS) analysis of a differential band in the SDS‐PAGE gel of Co‐IP products identified two histidine kinases encoded by the genes *COO91_09244* and *COO91_10729* (Table S1 and Figure S6B). Among these two putative interacting candidates, yeast two‐hybrid (Y2H) and *in vitro* pull‐down assays only confirmed an interaction between COO91_09244 and OrrA (Figure 5A,B). Unlike *orrA*, the expression of *COO91_09244* increased upon dehydration treatment (Figure S6C), and thus we designated COO91_09244 as Dsp1 (dehydration‐sensing protein 1). The structural models of Dsp1 and OrrA were predicted using AlphaFold 3 (https://alphafoldserver.com/), and their docking interaction was analyzed using ClusPro (https://cluspro.bu.edu/login.php). As shown in Figure 5C,D, Dsp1 primarily interacts with OrrA via its C‐terminal histidine kinase domain (Figure S6D).

**Figure 6:**
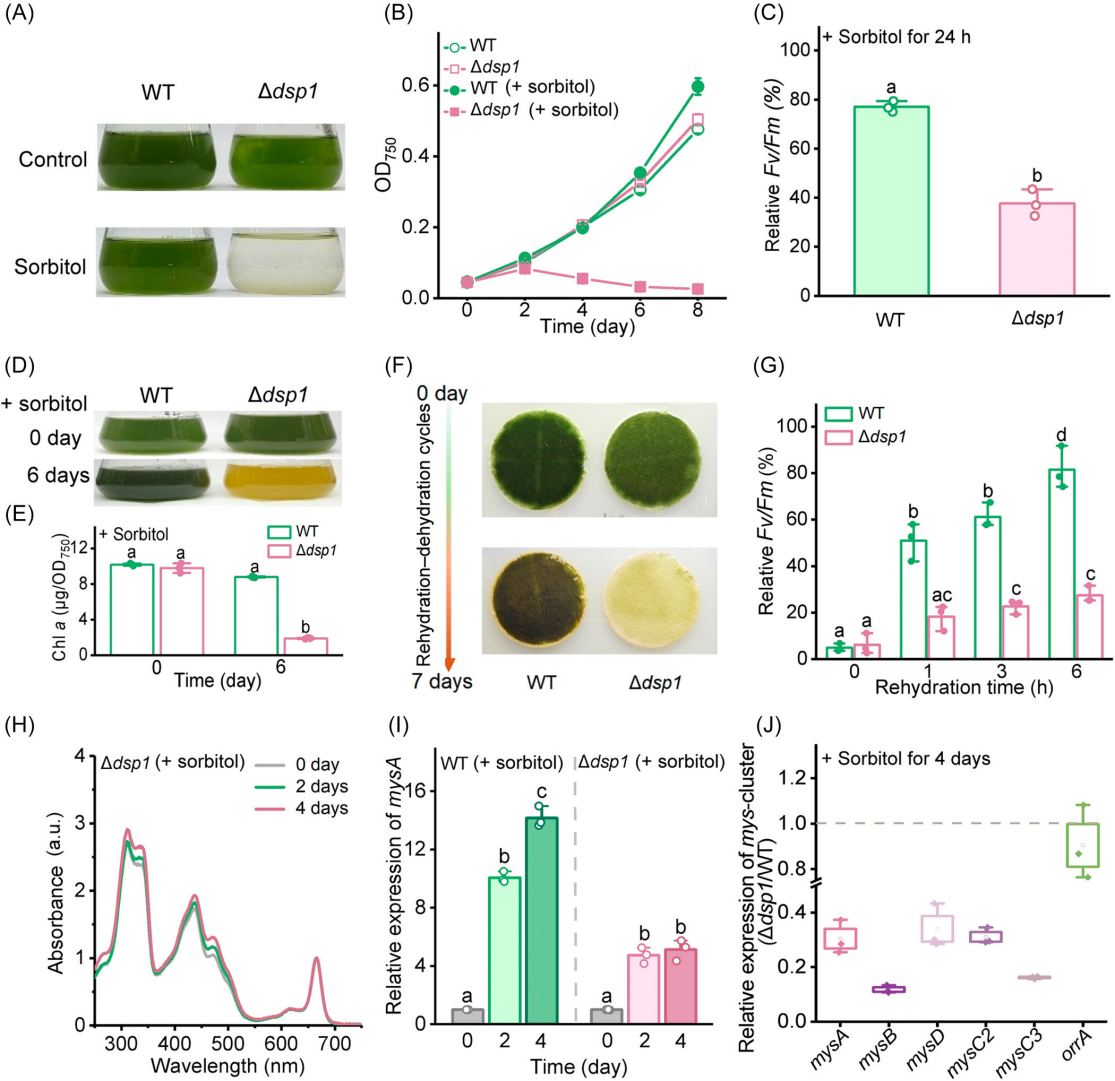
Dsp1 acts as an upstream regulator of OrrA and is involved in the regulation of MAA biosynthesis. (A, B) Comparison of growth between WT and Δ*dsp1* in BG11 medium. Cells were inoculated at an initial OD_750_ of 0.04 and grown under control conditions or with 0.3 M sorbitol to induce water‐deficit stress. Photographs were taken on Day 8 (A), with growth monitored every 2 days (B). (C) Comparison of PSII activity between WT and Δ*dsp1* after 24 h of treatment with 0.3 M sorbitol. Values are expressed relative to the initial measurements (set as 100%) taken at time zero, with baseline *Fv/Fm* values of 0.35 ± 0.01 for the WT and 0.31 ± 0.01 for Δ*dsp1*. (D, E) Comparison of chlorophyll *a* content between WT and Δ*dsp1* after 6 days of treatment with 0.3 M sorbitol. (F) Comparison of desiccation tolerance between WT and Δ*dsp1* following 1 week of rehydration–dehydration cycles. (G) Comparison of PSII recovery between WT and Δ*dsp1* after 7 days of rehydration–dehydration cycles. Values are expressed relative to the initial measurements (set as 100%) at time zero, with baseline *Fv/Fm* values of 0.37 ± 0.01 for WT and 0.33 ± 0.01 for Δ*dsp1*. (H) UV absorption spectra of MAAs in Δ*dsp1* after 4 days of treatment with 0.3 M sorbitol. Absorption peaks were normalized to chlorophyll *a* content. (I) Expression of *mysA* in WT and Δ*dsp1* after 4 days of treatment with 0.3 M sorbitol. (J) Transcript levels of the *mys* cluster and *orrA* in Δ*dsp1* after 4 days of treatment with 0.3 M sorbitol, relative to those in WT. Data are shown as the mean ± SD of three independent replicates. Different lowercase letters above the error bars indicate significant differences: for panels C, E, and G (*p* < 0.05, Student's *t*‐test) and for panel I (*p* < 0.05, Tukey's HSD).

To further investigate the biological role of *dsp1* in desiccation tolerance, we generated a *dsp1* knockout mutant (Δ*dsp1*) and evaluated its phenotype under dehydration. Under normal growth conditions, the growth rate of Δ*dsp1* was similar to that of WT strain, while upon exposure to 0.3 M sorbitol, Δ*dsp1* showed negligible biomass accumulation, whereas WT growth remained unaffected (Figure 6A,B). Correspondingly, after 24 h of 0.3 M sorbitol treatment, the residual *Fv/Fm* value of Δ*dsp1* (37.7% ± 4.5%) was significantly lower than that of the WT (77% ± 1.8%), indicating severe inhibition of PSII activity in the mutant (Figure 6C). Following 6 days of sorbitol treatment, Δ*dsp1* showed chlorosis (Figure 6D), with the chlorophyll *a* content reduced to only 21.44 ± 0.49% of WT levels (Figure 6E). When subjected to periodic desiccation on filter membranes over 7 days, Δ*dsp1* gradually bleached, while the WT strain remained largely unaffected (Figure 6F). After 7 days of dehydration–rehydration cycles, PSII recovery in Δ*dsp1* (27.39% ± 2.96%) was significantly lower than that in WT (81.48% ± 7.48%) following 6 h of rehydration (Figure 6G). These results demonstrate that loss of *dsp1* significantly compromises desiccation tolerance. Moreover, no significant induction of MAAs was detected in Δ*dsp1* under sorbitol treatment (Figure 6H), and the increase of the *mysA* expression was markedly reduced compared to WT (Figure 6I). After 4 days of sorbitol treatment, transcript levels of the *mys* cluster in Δ*dsp1* were substantially lower than those in the WT (Figure 6J), showing a pattern similar to that observed in Δ*orrA*. Taken together, these results suggest that Dsp1 functions in dehydration signal transduction and positively regulates MAA biosynthesis by activating the transcription factor OrrA.

## DISCUSSION

MAAs with broad taxonomic distribution have garnered prominent attention as potent UVR‐absorbing agents^12^. Accumulating evidence over the past decade further suggests that MAAs play additional roles as multifunctional secondary metabolites in diverse organisms^8^, ^39^. While direct functional evidence remains limited, several studies indicated a possible link between MAA production and habitat adaptability specifically in terrestrial cyanobacteria. Genomic analysis revealed a complete *mys* gene cluster in the desiccation‐tolerant cyanobacterium *N. commune*, in contrast to the desiccation‐sensitive species *N. verrucosum*, which lacks the *mysD* gene specifically^40^. Notably, a duplication event in the gene encoding MysC ATP‐binding ligase has been uniquely observed in desert‐dwelling cyanobacteria^29^. Despite these observations, the extent of MAA contribution to osmoprotection, the underlying molecular mechanisms, and the regulatory pathways remain largely unexplored. Here, using the desert cyanobacterium *N. flagelliforme* as a model, we provide genetic and physiological evidence demonstrating that MAAs function not only as antioxidants but also as effective osmolytes critical for cellular tolerance to dehydration stress (Figures 2 and 3). The MAA content in these cells reached approximately 2% of the cellular dry weight, which is substantially higher than the MAA levels previously reported in marine organisms^3^. The accumulation of MAAs in *N. flagelliforme* is comparable to that of classical osmolytes like trehalose and sucrose^41^, suggesting that MAAs can also serve as osmolytes for osmotic adjustment. Taken together, our findings support a critical role of MAAs as osmoprotectants in mediating desiccation tolerance in desert cyanobacteria.

Desiccation tolerance involves a highly coordinated suite of molecular adaptations^42^. During progressive dehydration, cells undergo transcriptional reprogramming and metabolic adjustment to enter an anhydrobiotic state. A hallmark of this adaptation is the accumulation of protective biomolecules, including specific proteins, sugars, and small solutes^43^. The well‐studied osmoprotectant trehalose protects cellular structures through water replacement, vitrification, and molecular shielding mechanisms^44^. Intrinsically disordered proteins such as LEA (late embryogenesis abundant) and CAHS (cytoplasmic‐abundant heat soluble) proteins synergize with trehalose by forming glassy matrices that stabilize macromolecules and membranes during dehydration^45^, ^46^. Critically, our data demonstrate that, at equivalent molar concentrations, MAAs provide slightly superior protection to LDH against desiccation‐induced damage compared to trehalose (Figure 3D), providing direct evidence of the dehydration‐stabilizing function of MAAs. Although the precise mechanism is still unclear, structural studies indicate that palythine, a representative MAA, forms a three‐dimensional hydrogen‐bonding network with water molecules^47^, ^48^, ^49^. Such networks may promote glass‐like matrix formation, reducing molecular mobility to prevent protein or membrane denaturation. The essential stabilizing role of MAAs during severe desiccation is further supported by the failure of the Δ*mysA* mutant (which lacks MAAs) to recover, in contrast to the WT strain that survives prolonged PEG‐induced stress and repeated dehydration–rehydration cycles (Figure 2G–I).

Desiccation induces excessive ROS production, posing a major challenge to cellular integrity, particularly in photosynthetic organisms like cyanobacteria, where ROS originates from both respiratory and photosynthetic pathways^50^. Consequently, enhanced antioxidant defense is essential for desiccation tolerance^51^, ^52^. While *in vitro* studies suggested potential antioxidant activity of MAAs^4^, ^53^, their *in vivo* roles in antioxidation remain poorly understood. Here, we demonstrate that dehydration triggers significantly higher accumulation of total ROS, singlet oxygen (¹O₂), and MDA in the Δ*mysA* mutant compared to the WT strain (Figures 3B,C and S5C). *In vitro* assays confirmed the ROS‐scavenging capacity of MAAs, revealing particularly strong activity against superoxide anions (O₂⁻) (Figures 3E,F and S5B). This defective antioxidation response likely explains the compromised post‐desiccation recovery observed in the Δ*mysA* mutant. In desiccation‐tolerant organisms, key antioxidation enzymes (e.g., superoxide dismutase, catalase, and peroxidase) often become inactivated during severe dehydration, increasing reliance on non‐enzymatic scavengers such as glutathione, tocopherols, and flavonoids^54^. We propose that MAAs play a parallel role as essential nonenzymatic antioxidants in cyanobacteria, complementing enzymatic defenses to enhance survival during desiccation. Although the precise chemical mechanism of MAA‐mediated ROS‐scavenging requires further studies, it likely involves direct radical neutralization or electron transfer to form less reactive species^49^.

The biosynthesis of compatible solutes is tightly controlled via signal transduction pathways. In *Cronobacter*, the EnvZ/OmpR two‐component system modulates trehalose production under osmotic stress^55^. Similarly, in *N. flagelliforme*, the red‐light signaling module NfPixJ–NfSrr1 plays a key role in regulating trehalose and sucrose biosynthesis during dehydration^41^. However, transcriptional regulation of MAA biosynthesis has received limited attention, with only a few regulatory factors (e.g., OrrA and Mig1) identified in certain cyanobacteria and fungi^28^, ^56^. Comprehensive studies on the upstream signal perception mechanisms and signaling pathways governing MAA production remain largely unexplored. In this study, we identified a dehydration‐responsive regulatory module comprising the transcription factor OrrA and its upstream dehydration‐inducible component Dsp1, which modulates MAA biosynthesis. Loss of either Dsp1 or OrrA impaired MAA induction under dehydration conditions and compromised desiccation tolerance (Figures 4 and 5). Strikingly, MAA induction by UVR remained largely unaffected in Δ*orrA* and Δ*dsp1* mutants (Figure S7), suggesting that the Dsp1–OrrA pathway is specifically dedicated to dehydration/osmotic regulation. Intriguingly, an OrrA homolog in *Nostoc* sp. PCC 7120 functions as a regulator of compatible solute synthesis under salt and drought stress^57^, ^58^. In the present study, the interaction between OrrA and the promoter of the sucrose synthase gene (*susB*) of *N. flagelliforme* was confirmed by yeast one‐hybrid (Y1H) assays (Figure S8A), suggesting that OrrA plays the same regulatory role in *N. flagelliforme*. Moreover, compared to the WT strain, the transcript levels of genes related to osmolyte synthesis, including *susA*, *susB*, and *treZ*, were significantly downregulated in Δ*orrA* and Δ*dsp1* mutants (Figure S8B,C). Transcriptional suppression of trehalose and sucrose biosynthesis in *N. flagelliforme* has been shown to reduce the accumulation of these compatible solutes^41^. Therefore, classical compatible solute levels would also be reduced in the Δ*orrA* and Δ*dsp1* mutants. These findings indicate that MAA biosynthesis regulation is an integral part of broader osmoregulatory networks controlling multiple osmolytes, further underscoring the critical role of MAAs as pivotal osmolytes.

Osmotic sensing is critical for survival in fluctuating environments. In bacteria, multiple two‐component systems (e.g., EnvZ/OmpR, MtrAB, and PhoQ/PhoP) sense osmotic changes via their sensor kinases' cytoplasmic or membrane‐associated domains in response to solute concentration or membrane perturbations^59^, ^60^, ^61^, ^62^. Precisely how these kinases detect such changes, however, remains unknown. In plants, mechanosensitive ion channels detect osmotic stress, initiating calcium influx and downstream signaling^63^. For example, the calcium‐permeable channel OSCA1, as an osmosensor, mediates stomatal closure under hyperosmotic stress^64^. More recently, DCP5 was identified as a cytoplasmic sensor for dehydration by forming osmostress granules that regulate gene expression^65^. By analogy, we propose that Dsp1 acts as a potential dehydration sensor in *N. flagelliforme*. Dsp1 contains two conserved period circadian protein (PAS) domains and a histidine kinase (HK) domain (Figure S6D). PAS domains function as ligand‐binding sensory modules that detect environmental cues. Their ligand‐induced conformational changes allosterically regulate the adjacent HK domain, thereby controlling autophosphorylation and downstream signaling^66^. PAS domains are present in sensor kinases that regulate responses to osmotic or redox signals^67^. Notably, a non‐light‐sensitive PAS domain in *Physcomitrella patens* mediates abscisic acid signaling, a central pathway for drought adaptation^68^. We therefore infer that Dsp1 perceives dehydration or redox changes via its PAS domains and transduces these signals by activating OrrA through a two‐component system. Dsp1‐mediated OrrA activation may involve phosphorylation of OrrA, which requires further study.

The scattered occurrence of MAA biosynthetic gene clusters among cyanobacteria is mirrored by the distribution patterns of other cyanobacterial secondary metabolite gene clusters, such as those for microcystin and scytonemin biosynthesis^28^, ^69^, ^70^. Our findings suggest that the MAA production via the *mys* biosynthetic pathway is an ancient cyanobacterial trait selectively conserved in lineages experiencing ecological desiccation stress (Figure S2). A specialized five‐gene *mys* cluster is consistently present in the genomes of terrestrial and symbiotic cyanobacterial strains from environments with episodic water availability, supporting a role for these MAA‐derived metabolites in water‐deficit tolerance. Notably, genes beyond the conserved core, such as *mysC3*, appear to be important for desiccation‐tolerant cyanobacteria but are not universally required among MAA producers. Phylogenetic analysis reveals a strong congruence between the *mys* gene cluster and species lineages (Figure S3). This pattern is most consistent with vertical inheritance alongside differential gene loss, although horizontal gene transfer cannot be ruled out. Similar to the evolutionary pattern reported for the microcystin biosynthesis gene cluster^69^, the *mys* cluster appears to be subject to purifying selection. This preserves its core functional modules across diverse taxa while allowing for lineage‐specific genomic rearrangements and adaptations. Notably, the cooccurrence of *dsp1* and *orrA* genes with this five‐gene *mys* cluster (Table S2) indicates that Dsp1–OrrA‐mediated MAA biosynthesis contributes to adaptation in water‐deficient environments. Collectively, these findings advance our understanding of how secondary metabolism facilitates cyanobacterial adaptation and persistence across diverse ecological niches.

The osmoprotective function of MAAs appears to be widely conserved across diverse organisms. In marine intertidal zones with daily desiccation fluctuations, dehydration‐induced salinity increase triggers a significant increase in MAA content in cyanobacteria like *Leptolyngbya* strain OU_13, indicating a positive correlation between MAA content and desiccation tolerance^71^. Our experimental data reveal that the Δ*mysC3* strain, which synthesizes only the MAA precursor 4‐DG, partially alleviates dehydration stress (Figure S4), suggesting that the conserved cyclohexenamine core structure itself may confer osmoprotective capacity. The role of MAAs in stress tolerance is critically dependent on stress‐responsive regulation and efficient biosynthesis. In *Nostoc*, the specialized Dsp1–OrrA module induces MAA accumulation specifically under water deficit. In contrast, MAA accumulation in marine organisms is regulated by multifactorial stressors, including salinity, UV radiation, and nutrient status^72^. This regulatory divergence is also evident in the structural variations of MAAs that facilitate adaptation: marine organisms primarily synthesize low‐molecular‐weight MAAs, such as shinorine, for rapid osmotic adjustment^8^, ^73^, ^74^, whereas terrestrial *Nostoc* produces complex derivatized MAAs to enhance desiccation tolerance. Thus, while the conserved MAA core structure likely provides fundamental osmoprotective capability, its variation and production rely on species‐specific regulatory systems and optimized biosynthetic efficiency.

**Figure 7:**
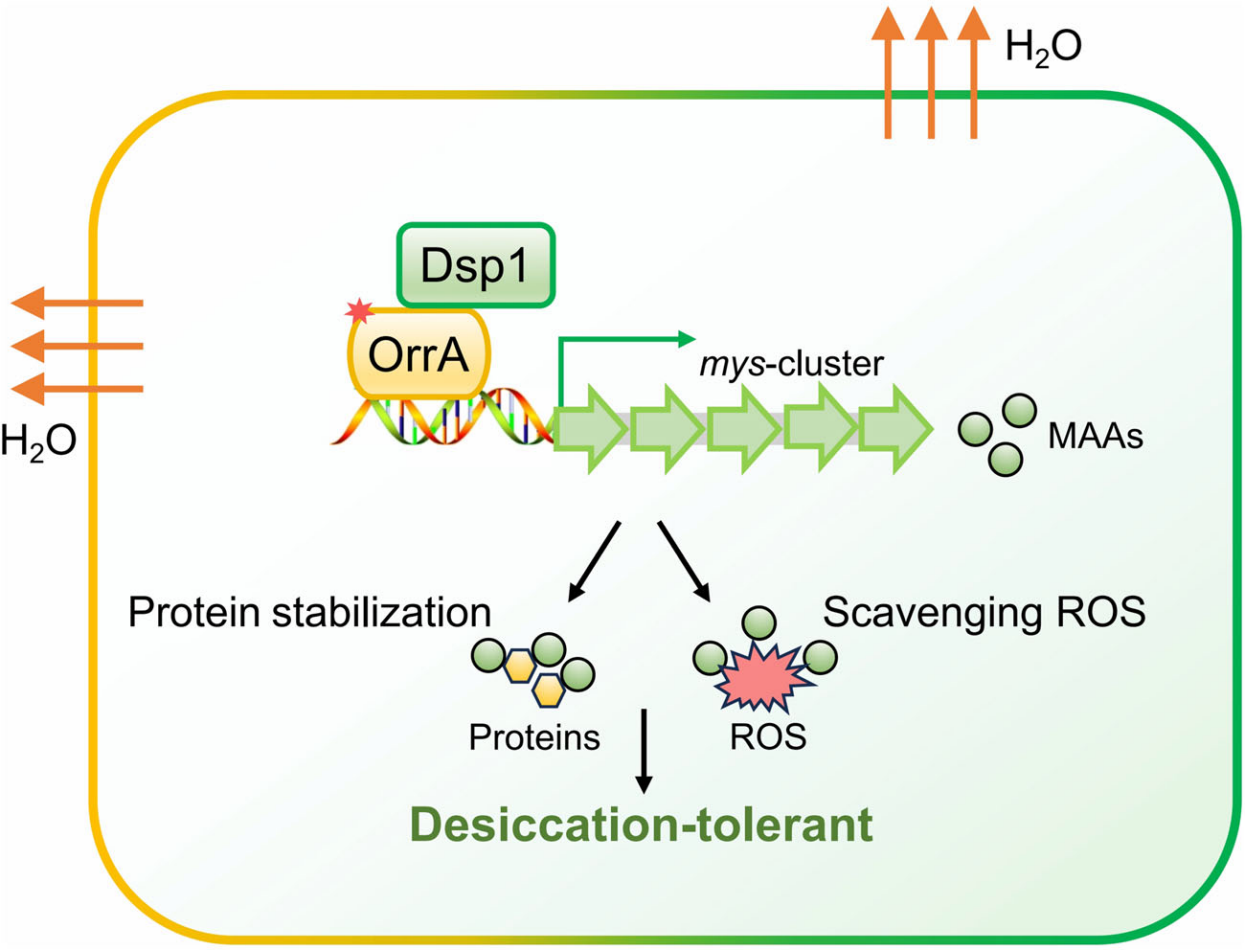
MAA‐mediated desiccation tolerance in *N. flagelliforme* through the Dsp1–OrrA pathway. The diagram summarizes how the Dsp1–OrrA regulatory module activates MAA production, which plays critical protective roles by stabilizing desiccation‐sensitive proteins and scavenging ROS. This mechanism highlights the importance of MAAs in the survival strategy of desert cyanobacteria under water‐deficit stress.

In conclusion, as illustrated in Figure 7, our findings establish a dual protective role for MAAs in the desert cyanobacterium *N. flagelliforme*, functioning as both osmolytes and antioxidants to enhance desiccation tolerance. We further identify the novel Dsp1–OrrA signaling module as a key regulatory pathway controlling MAA biosynthesis in response to water‐deficit condition. These findings provide novel molecular insights into MAA‐mediated desiccation tolerance, including the regulatory module for the dynamic tuning of MAA biosynthesis in response to dehydration stress. This work paves the way for engineering stress‐tolerant microbes and developing biotechnological applications for natural sunscreens.

## MATERIALS AND METHODS

### Cyanobacterial strains and culture conditions

The axenic strain *N. flagelliforme* CCNUN1 was isolated from dried samples collected from Sunitezuoqi, Inner Mongolia, China. The cyanobacterium *N. flagelliforme* was cultured at 25°C in BG11 medium in a test tube under continuous light (15 μmol photons m^−2^ s^−1^) and used for the measurements. *N. flagelliforme* cultures with an OD_750_ of 0.4 were treated with UV‐B (0.2 W m^−2^), 0.05 M NaCl, 0.3 M sorbitol, and 15% (wt/vol) PEG 6000. The contents of MAAs were detected on Day 0, 2, and 4.

### MAA extraction, purification, and detection

For MAA extraction and detection, 1 ml of cultures (OD_750_ adjusted to 0.4) was centrifuged at 13,000*g* for 5 min. The pellet was re‐suspended in 1 ml of 100% methanol and incubated at 4°C in the dark overnight. Following incubation, samples were centrifuged at 13,000*g* for 5 min and the supernatant was divided into two portions. One portion was used to measure the absorbance spectrum, recorded from 250 to 750 nm using a UV–visible spectrophotometer (UV2700; Shimadzu). The other portion was filtered through a 0.22 μm filter membrane and analyzed by HPLC (LC‐40; Shimadzu) with a photodiode‐array (PDA) detection at 310 nm.

For MAA purification, 80 ml of *N. flagelliforme* cultures (OD_750_ adjusted to 0.4) was harvested, and an equal volume of methanol was added to extract MAAs as described above. The supernatant was then collected for lyophilization over 16 h. The resulting dry powder was re‐dissolved in 16 ml of ultrapure water, and an equal volume of chloroform was added to remove pigments. After centrifugation (13,000*g*, 10 min), the upper aqueous phase was collected. Chloroform extraction was repeated twice to completely remove pigments. The final aqueous phase was filtered through a 0.22 μm filter membrane and analyzed by HPLC under the following conditions: column temperature 40°C, injection volume 150 μl, and mobile phase 20% methanol + 0.1% trifluoroacetic acid, run at a flow rate of 0.8 ml min^−1^. The detection wavelength was set at 310 nm. Fractions corresponding to the 8‐min peak were collected as purified MAAs.

### Total RNA isolation and RT‐qPCR analysis

30 ml of cultures (OD_750_ adjusted to 0.4) was harvested and centrifuged at 8000*g* for 10 min at room temperature. The samples were snap‐frozen and stored at −80°C. Total RNA was extracted using Trizol Reagent (Cwbio), and reverse transcription (RT) of RNA samples was performed using the PrimeScript FAST RT Reagent Kit (TaKaRa) following DNA removal. Transcript levels of *mysA*, *mysB*, *mysD*, *mysC3*, *mysC2*, *dsp1,* and *orrA* were quantified by RT‐qPCR (7900HT FAST Real‐Time PCR System, Applied Biosystems), using Taq Pro Universal SYBR qPCR Master Mix (Vazyme) and the primers from Table S3. The 16S rRNA gene served as the reference. The thermal cycling protocol consisted of 40 cycles of 10 s at 95°C, 30 s at 56°C, and 30 s at 72°C. Relative transcript levels were calculated using the $2^{-∆∆C_{t}}$ method^75^.

### Construction of mutants and stress treatments

CRISPR‐Cpf1 genome editing technology was used to generate specific gene deletions in *N. flagelliforme* as previously described^31^. The protospacer adjacent motif (PAM) for the Cpf1 is a 5′T‐rich PAM (5′‐TTN‐3′), and 22 nt spacer sequences were designed for the CRISPR array, which was cloned into a pCpf1 vector. The flanking sequences approximately 800–1000 bp in size surrounding the target of interest were amplified by PCR and fused by overlapping PCR and the primers listed in Table S3. The pCpf1 plasmid was linearized using *Bam*HI and ligated with the recombinant gene fragment through homologous recombination, then transferred into *Escherichia coli* HB101. The constructed plasmid was subsequently transferred into *N. flagelliforme* using the conjugal transfer method. The mutants were selected on 1.5% agar BG11 plates containing 30 μg ml^−1^ spectinomycin under 15 μmol photons m^−2^ s^−1^. Positive single clones were verified by PCR (Figure S9) and sequencing. After several subcultures, cells were plated onto solid medium containing 5% sucrose to allow the loss of the editing plasmids. All strains used in this study are listed in Table S4.

For stress treatments, 100 ml of *N. flagelliforme* cultures (OD_750_ of 0.4) was collected and re‐suspended in an equal volume of liquid BG11 medium containing 0.3 M sorbitol, 15% PEG 6000, or 0.05 M NaCl at 25°C under continuous 15 µmol photons m^−2^ s^−1^ white fluorescent light. For UV‐B treatment, *N. flagelliforme* cultures were exposed to 0.2 W m^−2^ UV‐B. All treatments were started at an initial OD_750_ of 0.4, and samples were collected every 2 days for absorbance measurement between 250 and 750 nm. To quantify MAAs as a percentage of cellular dry weight in *N. flagelliforme*, MAAs were extracted from 1 ml of culture using methanol. The MAA concentration was determined by measuring the absorbance at 310 nm and applying the formula *c* (mg ml^−1^) = *A*/(*ε* × *l*) × MW^76^, where *A* represents the average absorbance, *ε* is the molar extinction coefficient, *l* is the path length (1 cm), and MW is the molecular weight. This concentration was converted into mass per gram dry weight (mg g^−^¹ DW) based on the dry biomass of the 1 ml sample. The percentage of MAAs relative to cellular dry weight was then calculated as [(mg g⁻¹ DW)/1000] × 100%. For growth monitoring under 0.3 M sorbitol and 15% PEG 6000 treatments, all cultures were initiated at an OD_750_ of 0.04. The growth of cyanobacterial cells was monitored every 2 days by measuring the OD_750_ using a spectrophotometer, and photographs were taken on the eighth day. Cultures grown in normal BG11 medium served as the control.

### Determination of chlorophyll fluorescence and photosynthetic oxygen evolution rate

*N. flagelliforme* cultures with an OD_750_ of 0.4 were re‐suspended in BG11 medium containing 0.3 M sorbitol, and then cultured under 15 µmol photons m^−2^ s^−1^. The maximal quantum yield of PSII (*Fv/Fm)* were monitored at 0, 4, 8, 16, and 24 h using Handy Plant Efficiency Analysis (Hansatech Instruments). Photosynthetic oxygen evolution was determined at 0 and 48 h using the Clark‐type oxygen electrode (Chlorolab 2, Hansatech Instruments).

### Electrolyte leakage assay

REC was measured according to a previously described method^77^. 5 ml of the *N. flagelliforme* cultures was harvested by centrifugation at 7000*g* for 5 min at room temperature, then resuspended in 10 ml ultrapure water, and incubated for 10 h at room temperature. Initial conductivity (*C*_t_) was measured using the electrical conductivity meter (AZ Instrument). Then, the samples were boiled for 20 min, cooled to 25°C, and the final conductivity (*C*_k_) was recorded. REC was calculated as (*C*_t_/*C*_k_) × 100%. Finally, the relative change of REC was represented by the difference between the REC values after and before the stress treatment.

### Determination of MDA content

MDA levels were determined using the thiobarbituric acid (TBA) method^78^. 5 ml of *N. flagelliforme* cultures was centrifuged at 4000*g* for 10 min. The pellets were then resuspended in 2 ml of 10% trichloroacetic acid (TCA) solution and ground into a powder. After thorough grinding, the samples were transferred to centrifuge tubes. After centrifuging at 4000*g* for 10 min, 2 ml of the supernatant was collected. Then, 2 ml of a 0.6% TBA solution was added to the supernatant, with 2 ml of distilled water as a blank. The mixture was boiled for 15 min, and then rapidly cooled on ice for 5 min and centrifuged. The optical density of the supernatant was measured using a UV‐visible spectrophotometer (UV2700; Shimadzu) at 532, 600, and 445 nm. The content of MDA was calculated using the following formulas: *C* (μmol/OD_750_) = [6.45 (*A*_532nm_ – *A*_600nm_)−0.56*A*_450nm_]/OD_750_.

### Determination of intracellular ROS

Intracellular ROS levels in *N. flagelliforme* were measured using the cell‐permeant tracer 2’,7’‐dichlorofluorescein diacetate (DCFH‐DA) as previously described^31^. Cells were centrifuged at 13,000 *g* for 10 min and resuspended in 2 ml of TE buffer (10 mmol l^−1^ Tris‐base, 1 mmol l^−1^ EDTA‐Na_2_, 0.5 mmol l^−1^ NaCl, pH 8.0). Cells were then incubated with 10 μmol l^−1^ DCFH‐DA (Sigma Aldrich Inc.) at 25°C for 1 h in the dark. After incubation in the dark, cells were washed twice with fresh medium to remove extracellular DCFH‐DA. DCFH‐DA can easily penetrate the cellular membrane and is cleaved by intracellular esterases. This biochemical reaction transforms DCFH‐DA into the nonfluorescent compound DCFH, which is then rapidly oxidized to highly fluorescent 2′,7′‐dichlorofluorescein (DCF) in the presence of ROS. Therefore, the fluorescence signal of DCF represents the amounts of ROS. Fluorescence intensity was measured with a Hitachi F‐4700 fluorescence spectrophotometer at excitation 488 nm and emission 525 nm.

### Measurement of radical‐scavenging activity of MAAs

The scavenging capacity of purified MAAs on a superoxide anion (O_2_^–^), a DPPH radical, and ABTS radical cations (ABTS•^+^) was determined using the superoxide anion‐scavenging ability detection kit (BC4770; Solarbio), DPPH assay kit (BC4750, Solarbio), and the ABTS assay kit (BC4770; Solarbio), respectively. The O_2_^−^‐scavenging activity of MAAs was assessed at concentrations of 4, 8, and 12 mM according to the manufacturer's protocol. The concentration of MAAs used for the ABTS and DPPH radical‐scavenging ability assays was 2.6 mM.

### LDH enzyme activity assay

LDH activity assays were performed according to a published protocol^24^. The LDH enzyme from rabbit muscle (Roche, Aldrich Chemical Company) was prepared by diluting it to a concentration of 0.025 g l^−1^ in 200 μl of 25 mM Tris/HCl buffer (pH 7.0), with three groups: one containing 30 mM of MAAs, one containing the same concentration of d‐trehalose, and a control group without any additional agents. Half of each sample was kept at 4°C to serve as the untreated control, while the other portion was subjected to dehydration in a vacuum concentrator system at 25°C overnight. After complete desiccation, the samples were rehydrated with 250 μl of deionized water. All samples were kept on ice until their enzyme activity was determined. To assess LDH activity, 10 μl of the samples was mixed with 990 μl of 100 mM sodium phosphate buffer (pH 6.0), which contained 100 mM NADH and 2 mM pyruvate. Absorbance at 340 nm was measured every 1 s for a duration of 1 min using a UV–visible spectrophotometer (UV2700; Shimadzu). Relative activity was calculated based on the untreated control. Each experiment was repeated three times.

### Y2H assay

The interactions between Dsp1 and OrrA were performed using the Matchmaker GAL4 Two‐Hybrid System 3 (Clontech). These genes were amplified by PCR using genomic DNA from *N. flagelliforme* CCNUN1 as the template with high‐fidelity Pfu DNA polymerase (Promega). The primers are presented in Table S3. The fragments of *orrA* and *dsp1* were cloned into pGADT7 and pGBKT7 vectors (Clontech), respectively. The purified pGADT7‐OrrA and pGBKT7‐Dsp1 plasmids were co‐transformed into the host strain *Saccharomyces cerevisiae* AH109. Transformants were selected on an SD/‐Trp‐Leu agar plate, and the interaction was assessed by growing the transformants on SD/‐Trp/‐Leu/‐His agar plates and incubating for 3 days.

### Y1H assay

The Y1H assay enables sensitive detection of a protein's intrinsic DNA‐binding capacity at the level of physical interaction^79^. Y1H assays were performed as described previously^41^. Briefly, the promoter region of *susB* (*pro‐susB*) was recombined into the yeast reporter vector pAbAi, and the plasmid was transformed into the Y1H Gold yeast strain. Then, the purified pGADT7‐OrrA plasmid was transformed into the yeast bait Y1H Gold strain carrying the pAbAi‐*pro‐susB* construct, which was used to verify its interaction with *pro‐susB* segment. The pGADT7 empty vector was used as a negative control. Transformants were selected and confirmed on the SD plates minus leucine with 425 μg l^−1^ aureobasidin A (AbA) for 3 days to verify the binding of transcription factor OrrA with the *susB* promoter. Primers are listed in Table S3.

### Pull‐down assay

The pull‐down assay was performed as described previously^35^. The *dsp1* and *orrA* genes were respectively cloned into the pGEX‐4T‐1 and pET‐28a vectors using the primers listed in Table S3. The recombinant plasmids were transformed into *E. coli* BL21 (DE3) to express the fusion proteins GST‐tagged Dsp1 (GST‐Dsp1) and His‐tagged OrrA (His‐OrrA). Cells expressing these fusion proteins were lysed, and the cell lysates were centrifuged at 13,000*g* for 1 h. The crude protein extract was subsequently incubated on ice overnight to allow the protein to fully interact with the target proteins. Pull‐down assays were performed using GST resins. The protein interactions between Dsp1 and OrrA were assessed using GST‐tagged Dsp1 with His‐OrrA, with GST as a negative control to exclude non‐specific interactions. Protein interactions were detected by Western blotting using anti‐His and anti‐GST antibodies.

### Co‐IP assay and LC‐MS/MS

The Co‐IP assay was modified from a previously described method^80^. *N. flagelliforme* cells were harvested by centrifugation at 5000*g* for 20 min at 4°C. The pellet was washed and resuspended in ice‐cold KPN buffer (40 mM potassium phosphate, pH 8.0, 100 mM NaCl, 1 mM DTT, and 100 μg ml^−1^ PMSF). The cell suspension was lysed, and the lysate was centrifuged at 9000*g* for 1 h at 4°C to collect the soluble protein fraction. The resulting pellet was re‐suspended in ice‐cold HEPES buffer (1.2 M betaine, 5% glycerol, 100 mM NaCl, 5 mM MgCl₂, 10 μM ZnCl₂, 1 mM DTT, 0.05% β‐DM, pH 7.2) and incubated on ice for 30 min to slowly solubilize membrane proteins.

For immunoprecipitation, 500 μl of total protein extract was incubated with 1 μg of the anti‐OrrA antibody or a corresponding control IgG for 1 h at 4°C with gentle rotation. Subsequently, 20 μl of protein A/G agarose beads (4% cross‐linked) was added, and the mixture was incubated overnight at 4°C to capture immune complexes. The sample were then centrifuged at 10,000*g* for 5 min at 4°C. The pellet was washed three times with Buffer Z (25 mM HEPES‐KOH, pH 7.8, 12.5 mM MgCl₂, 1 mM DTT, 20% glycerol), and the supernatant was removed after each wash. Finally, the pellet was re‐suspended in 40 μl of 1× SDS loading buffer and boiling for 5 min. The eluted proteins were separated by SDS‐PAGE, and specific bands of interest were excised for identification by LC‐MS/MS using the UniProt database (https://www.uniprot.org/) for protein matching.

### Determination of intracellular ^1^O_2_ generation

A singlet oxygen detection kit (BestBio, BBoxiProbe® O66) was used to detect the generation of singlet oxygen (^1^O_2_) in *N. flagelliforme*. The sample treatment and fluorescence intensity measurement were performed as described for intracellular reactive oxygen species determination, with the exception that the reaction system included the ^1^O_2_ fluorescent probe.

### Measurement of ^1^O_2_‐scavenging activity and hydroxyl radical (·OH)

Singlet oxygen (^1^O_2_)‐scavenging ability of MAAs was assessed based on the changes in singlet oxygen content. The generation of ^1^O_2_ *in vitro* and the determination of scavenging ability were performed according to the method described previously^81^, and the ^1^O_2_ contents were determined using the singlet oxygen detection kit (BestBio, BBoxiProbe® O66). The scavenging capacity of purified MAA on a hydroxyl radical (·OH) was determined using the hydroxyl radical‐scavenging ability detection kit (Solarbio, BC1320). In brief, the MAAs diluted to concentrations of 0.5, 1.0, and 2.0 mM, and the scavenging capacity was determined according to the kit instructions.

### Phylogenetic analyses

Putative MAA biosynthetic proteins were identified by BLASTp search against the NCBI non‐redundant protein database, using MysA and MysB from *N*. *flagelliforme* CCNUN1 as initial queries. Candidate MAA biosynthetic gene clusters were retrieved based on protein cluster information available in NCBI. The 16S rRNA gene sequences and selected protein sequences were aligned using MAFFT v7, and the resulting alignments were visualized with ESPript 3.0^82^. Phylogenetic analyses were performed with PhyML 3.0 using automatic model selection^83^. The best‐fit evolutionary models (GTR + G + I + F for nucleotides and WAG + G + I + F for proteins) were determined and evaluated with 1000 bootstrap replicates. Finally, the 78 cyanobacterial strains were annotated on the phylogenetic tree, with colored dots corresponding to their respective habitats. The methodology used to investigate the occurrence of the *mys* gene cluster among desiccation‐tolerant *Nostoc* strains was adapted from approaches previously used to describe a similar evolutionary pattern for the microcystin biosynthesis gene cluster^69^.

### Statistical analysis

The software Origin 2017® was used for graphing, and IBM SPSS 28.0® for statistical analysis. For all assays, at least three independent biological replicates were performed unless indicated otherwise. Comparisons were made using one‐way ANOVA, and when significant differences were found, the Tukey test was applied for post hoc analysis (*p* < 0.05). Comparisons between two variables were performed using a unpaired Student's *t*‐test (*p* < 0.05). The results are presented as the mean ± standard deviation (SD).

## AUTHOR CONTRIBUTIONS

**Lei Zhao**: Formal analysis; investigation; writing—original draft; writing—review and editing. **Hai‐Feng Xu**: Conceptualization; formal analysis; funding acquisition; investigation; writing—original draft; writing—review and editing. **Jin‐Long Shang**: Investigation. **Yong Li**: Investigation. **Xiao‐Yue Yin**: Investigation. **Zhong‐Chun Zhang**: Investigation. **Huazhong Shi**: Formal analysis; writing—original draft; writing—review and editing. **Bao‐Sheng Qiu**: Conceptualization; formal analysis; funding acquisition; supervision; writing—original draft; writing—review and editing.

## ETHICS STATEMENT

No animals or humans were involved in this study.

## CONFLICT OF INTERESTS

The authors declare no conflict of interests.

## Supporting information

Supporting Information.

## Data Availability

All data needed to evaluate the conclusions of this study are present in the paper and the Supplementary Materials.
