# Supplementary material for: Osmotic signaling governs sunscreen biosynthesis to safeguard desert cyanobacteria against desiccation
Source: mLife. 2026 Apr 10;5(2):164–79. doi: 10.1002/mlf2.70075 (PMC13131328; doi:10.1002/mlf2.70075)
Supplement: Supplementary file 1 — Supporting Information. [file MLF2-5-164-s001.pdf]

Figure S1

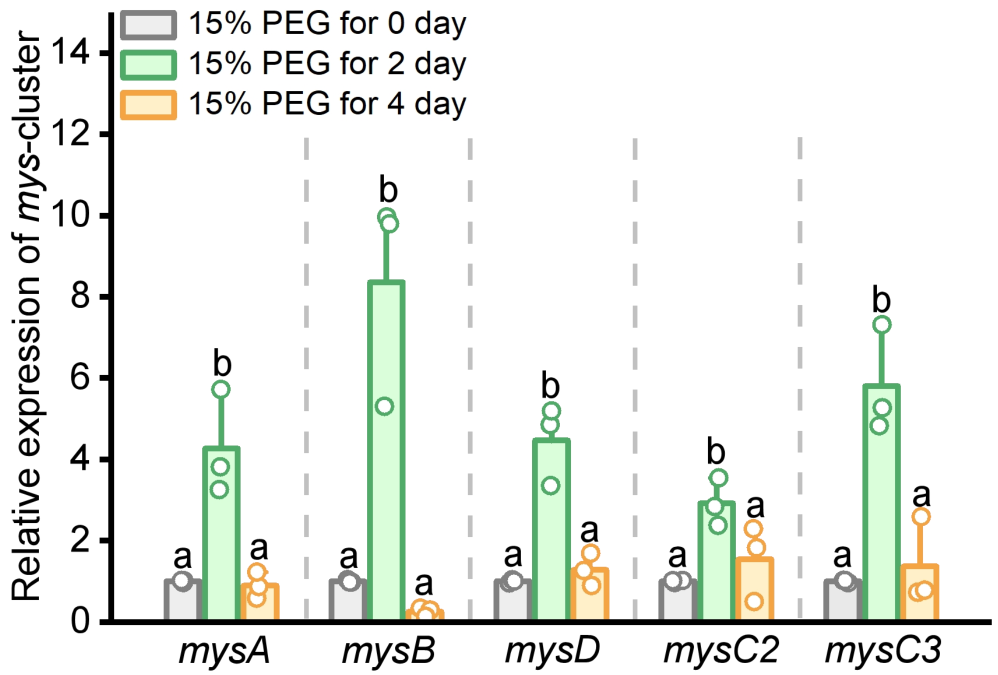

Figure S1. Relative transcript levels of the *mys*-cluster in the WT strain following treatment with 15% (wt/vol) PEG 6000 over time.

Data are shown as the mean ± SD of three independent replicates. Different letters above the error bars indicate significant differences ($p < 0.05$, Tukey's HSD).

Figure S2

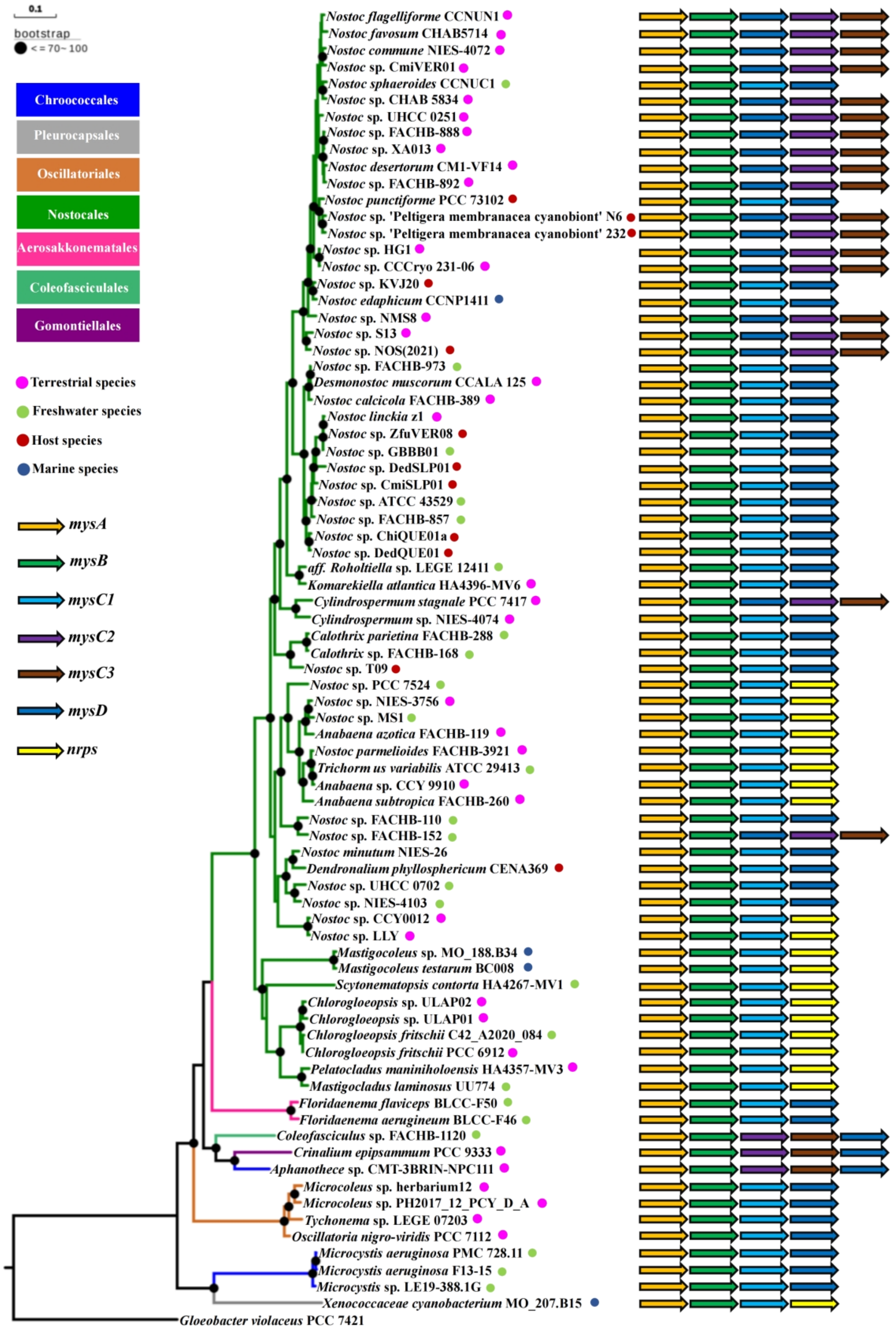

Figure S2. Evolutionary relationship analysis between the five-gene *mys* cluster involved in MAA biosynthesis and desiccation-tolerant cyanobacteria.
Maximum-likelihood phylogeny of cyanobacteria based on 16S rRNA gene sequences and genetic organizations of the putative MAA biosynthesis gene cluster. Branches are color-coded according to the classification of orders. *Gloeobacter violaceus* PCC 7421 was set as the outgroup. Black dots indicate that the nodes supported with a bootstrap of ≥70%.

Figure S3

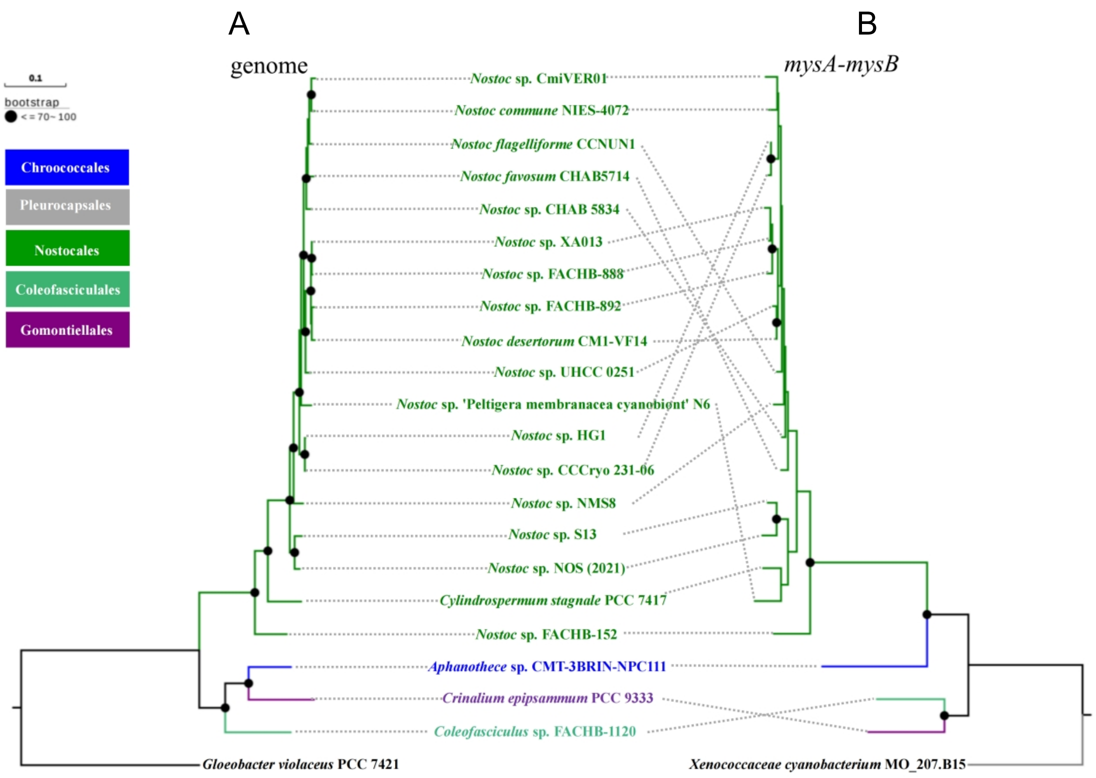

Figure S3. Coevolution of the five-gene *mys* cluster with desiccation-tolerant strains of *Nostoc*.

(A) The maximum-likelihood tree was generated by concatenating the dataset of 31 conserved proteins. (B) The maximum-likelihood tree based on the *mysA-mysB* data set. Branches are color-coded according to the classification of orders. *Gloeobacter violaceus* PCC 7421 and *Xenococcaceae* cyanobacterium MO_207.B15 were set as the outgroup, respectively. Black dots indicate that the nodes supported with a bootstrap of ≥70%.

Figure S4

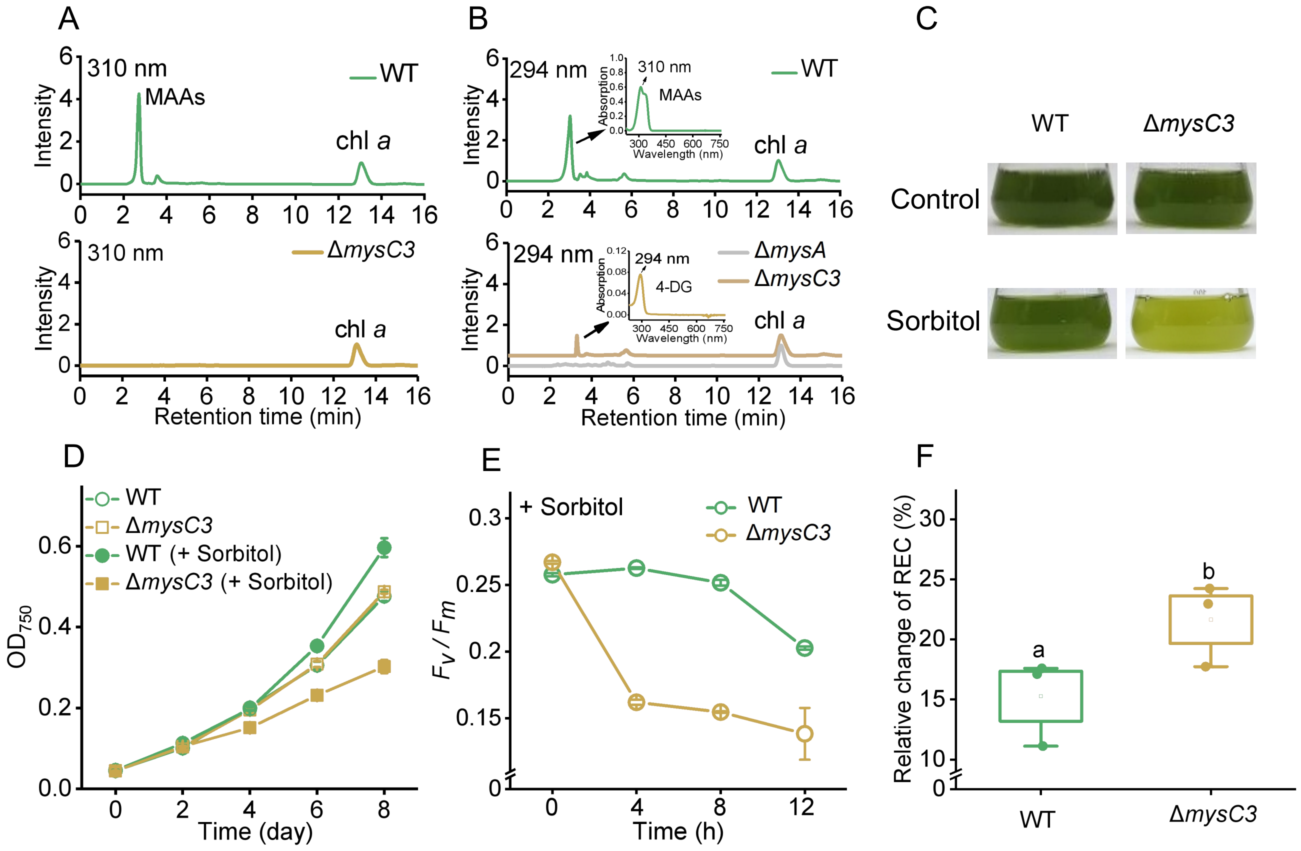

Figure S4. Deletion of *mysC3* reduces the tolerance of *N. flagelliforme* to water-deficit stress.

(A) HPLC chromatograms of methanolic extracts from the WT and Δ*mysC3* with UV absorption at 310 nm.

(B) HPLC chromatograms of methanolic extracts from the WT, Δ*mysA* and Δ*mysC3* with UV absorption at 294 nm. The inset shows the online UV absorption spectra of the characteristic peaks from WT (2.8 min) and Δ*mysC3* (3.3 min).

(C-D) Growth comparison between WT and Δ*mysC3* in BG11 medium. Cells were inoculated at an initial OD_{750} of 0.04 and grown under control conditions or with 0.3 M sorbitol to induce water-deficit stress. Photographs were taken on Day 8 (C), and growth was monitored every two days (D).

(E) Effects of short-term 0.3 M sorbitol treatment on *Fv/Fm* in the WT and Δ*mysC3*.

(F) Changes in REC levels in the WT and Δ*mysC3* after 2 days of exposure to 0.3 M sorbitol, normalized to their respective untreated controls.

Data are shown as the mean ± SD of three independent replicates. Different letters above the error bars in panel F indicate significant differences ($p < 0.05$, Student's *t*-test).

Figure S5

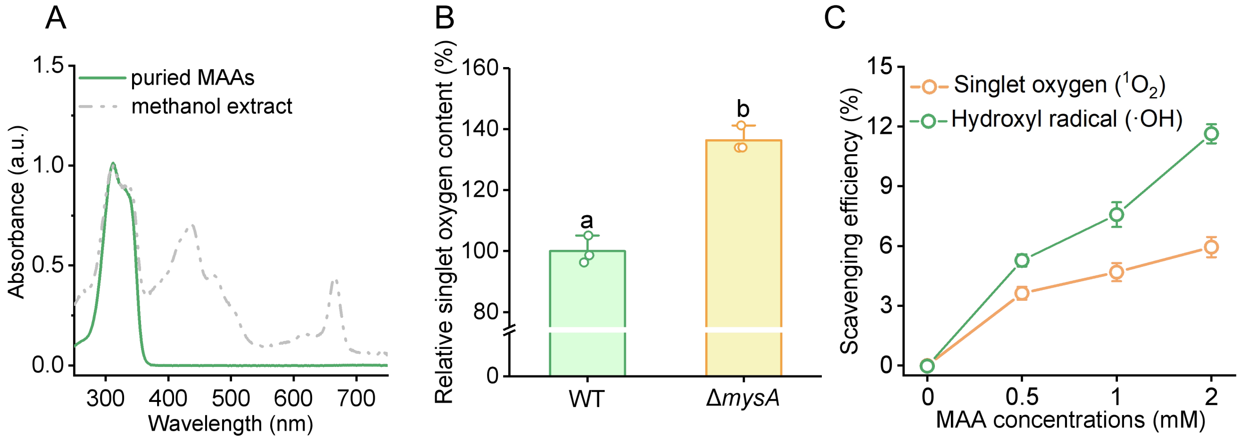

Figure S5. *In vitro* antioxidant activity of MAAs from *N. flagelliforme*.

(A) UV-Vis absorption spectra of methanolic extracts (dotted line) from *N. flagelliforme*, and purified MAAs (solid line) dissolved in water.

(B) The basal levels of singlet oxygen ($^1O_2$) in the Δ*mysA* are higher than those in the WT.

(C) Dose-dependent scavenging capacity of MAAs against hydroxyl free radical (·OH) and singlet oxygen ($^1O_2$).

Data are shown as the mean ± SD of three independent replicates. Different letters above the error bars in panel B indicate significant differences (p < 0.05, Student's t-test).

Figure S6

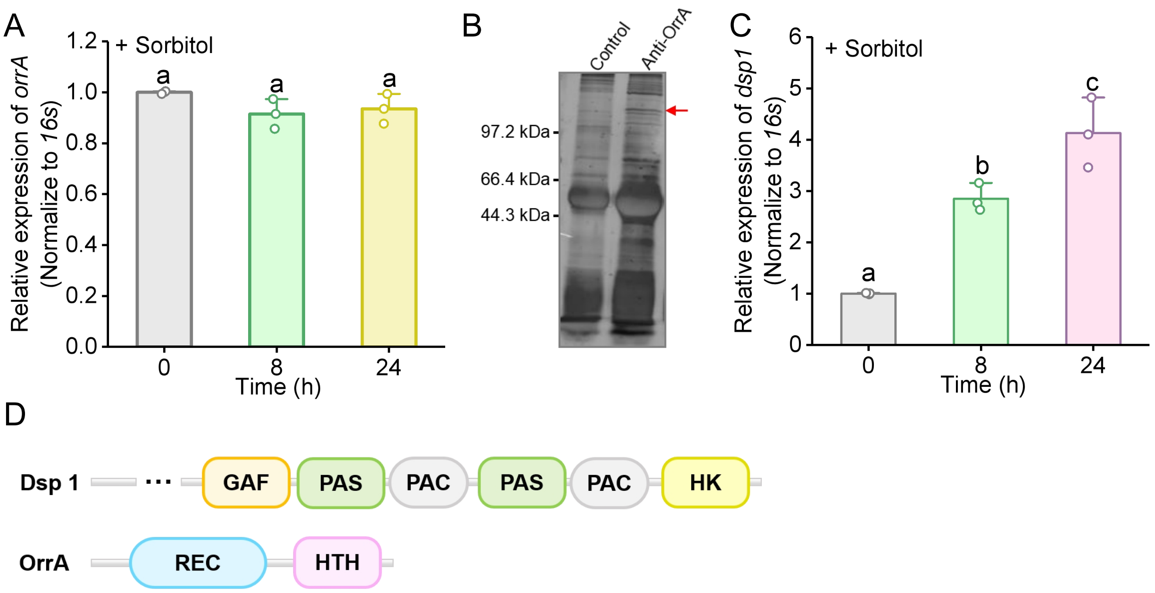

Figure S6. Identification of Dsp1 and its positive response to dehydration.

(A) Transcription levels of *orrA* (normalized to 16S rRNA) in *N. flagelliforme* under 0.3 M sorbitol treatment for 0, 8 and 24 hours, expressed relative to the 0-hour time point.

(B) SDS-PAGE analysis of Co-IP samples for anti-OrrA antibody immunoprecipitation. Lane 1: negative control (Co-IP with rabbit pre-immune serum). Lane 2: experimental sample (Co-IP with a custom rabbit polyclonal antibody against OrrA). The red arrow indicates the specifically enriched band in the experimental lane. Protein identification by LC-MS of the differential band excised from the SDS-PAGE gel of Co-IP products is summarized in Table S1.

(C) Transcription levels of *dsp1* (normalized to 16S rRNA) in *N. flagelliforme* under 0.3 M sorbitol treatment for 0, 8 and 24 hours, expressed relative to the 0-hour time point.

(D) Protein domains of Dsp1 and OrrA predicted by SMART (http://smart.embl.de/). GAF, cGMP phosphodiesterase adenylyl cyclase and FhlA domain; PAS, period circadian protein; PAC, C-terminal to PAS motifs; HK, histidine kinase with ATPase; REC, receiver domain; HTH, helix-turn-helix domain.

Data are shown as the mean ± SD of three independent replicates. For panels A and C, different letters above the error bars indicate significant differences ($p < 0.05$, Tukey's HSD).

Figure S7

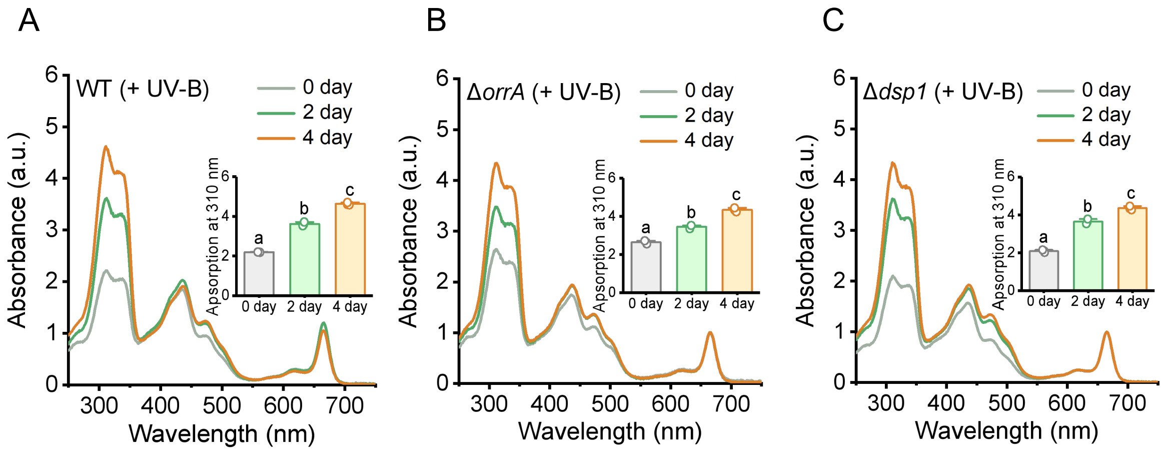

Figure S7. Comparative analysis of MAA biosynthesis induced by UV-B in the WT, *ΔorrA* and *Δdsp1* strains.

(A-C) The UV absorption spectra of MAAs in the WT (A), Δ*orrA* (B) and Δ*dsp1* (C) strains treated with 0.2 W m^{-2} UV-B for 0, 2, and 4 days, respectively. The absorption peaks of each sample were normalized to chlorophyll *a* content.

Data are shown as the mean ± SD of three independent replicates. Different letters above the error bars indicate significant differences ($p < 0.05$, Tukey's HSD).

Figure S8

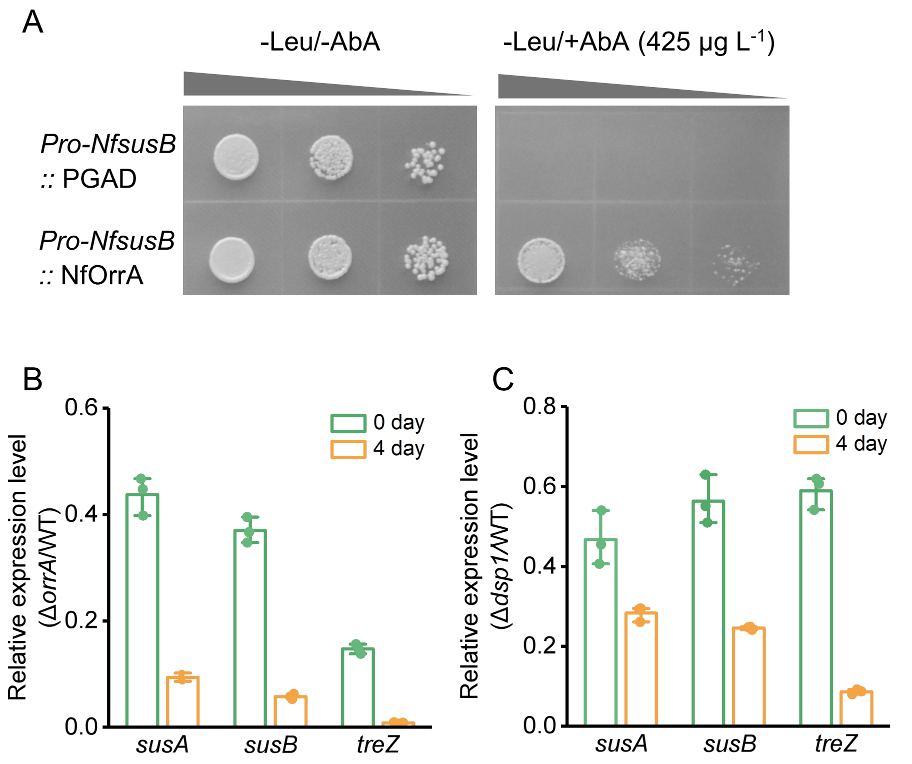

Figure S8. Dsp1-OrrA signaling module positively regulates the genes associated with osmolyte synthesis.

(A) *In vitro* interaction between transcription factor OrrA and the promoter of *susB* gene encoding sucrose synthase by yeast one-hybird assay.

(B-C) Relative transcription levels of *susA*, *susB*, and *treZ* in Δ*orrA* and Δ*dsp1* treated with 0.3 M sorbitol for 0 and 4 days, normalized to the corresponding levels in the WT. Data are shown as the mean ± SD of three independent replicates.

Figure S9

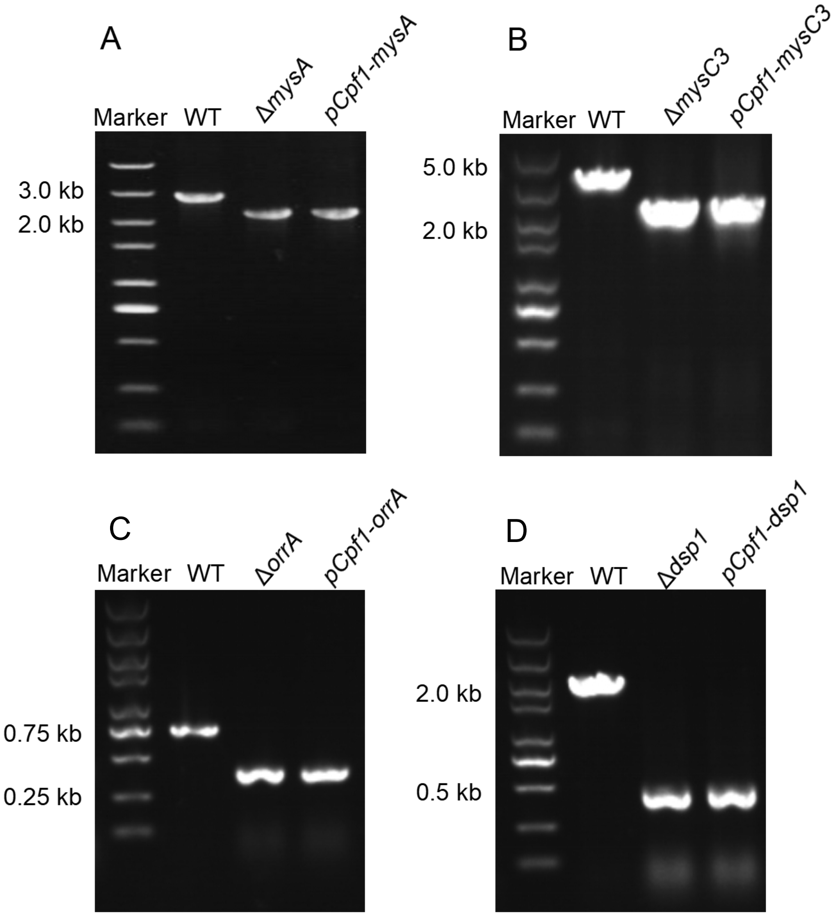

Figure S9. PCR-based identification of *N. flagelliforme* CCNUN1 mutants.
Genomic DNA from the WT strain, each mutant strain, and the corresponding plasmid (pCpf1-target gene) was used as the template for PCR analysis. Panels A-D show the identification results for the Δ*mysA*, Δ*mysC3*, Δ*orrA* and Δ*dsp1* mutants, respectively. The specific PCR primers used for mutant verification are listed in Table S3.

Table S1. Identification of proteins from the differential band in the SDS-PAGE gel of Co-IP products (shown in Fig. S6B) using LC-MS and protein database searching.

Gene name	Annotation	Score	MW (kDa)
COO91_03993	rpoB, DNA-directed RNA polymerase subunit beta	3018	123
COO91_02736	carB, carbamoyl-phosphate synthase large subunit	180	119
COO91_01514	cpcA, phycocyanin alpha chain	148	17
COO91_04977	dnaK, molecular chaperone DnaK	87	72
COO91_01515	cpcB, phycocyanin beta chain	80	18
COO91_03780	apcA, allophycocyanin alpha subunit	73	17
COO91_03995	rpoC, DNA-directed RNA polymerase beta subunit	62	147
COO91_04899	5-methyltetrahydrofolate--homocysteine methyltransferase	60	131
COO91_06383	Carbohydrate-selective porin OprB	54	61
COO91_02701	infB, translation initiation factor IF-2	48	99
COO91_02158	Serine/threonine protein phosphatase PrpC	48	28
COO91_04602	photosystem I core protein PsaB	47	83
COO91_05535	Signal transduction histidine kinase	37	83
COO91_01951	Glutamate-1-semialdehyde aminotransferase	37	219
COO91_05369	Histidine kinase-like ATPase, C-terminal domain	35	43
COO91_01397	transposase	33	13
COO91_02456	msmX, multiple sugar transport system ATP-binding protein	33	41
COO91_03953	kojP, kojibiose phosphorylase	33	91
COO91_03299	histidine kinase	33	75
COO91_08120	ABC-type bacteriocin/lantibiotic exporters	31	105
COO91_03781	apcB, allophycocyanin beta subunit	31	17
COO91_04250	putative Zn-dependent peptidase	30	46
COO91_09461	putative enzyme involved in methoxymalonyl-ACP biosynthesis	28	41
COO91_08703	histidine kinase	28	205
COO91_07052	Small ligand-binding sensory domain FIST	28	48
COO91_06940	Serine/threonine protein kinase	26	64
COO91_06533	F-type H+-transporting ATPase subunit beta	26	52
COO91_05248	histidine kinase	25	56
COO91_08417	Phage DNA replication protein O	24	56
COO91_06354	Heme-degrading monooxygenase HmoA	23	5
COO91_06616	RP-L17, large subunit ribosomal protein L17	23	13
COO91_07366	putative protein family, nitrogen fixation	21	9
COO91_09264	Serine protease, subtilisin family	19	47
COO91_01855	Formylglycine-generating enzyme	18	106
COO91_06179	Replication-associated recombination protein	18	13
COO91_06255	DNA repair exonuclease ATPase subunit	17	98
COO91_10729	two-component sensor histidine kinase	17	233
COO91_09244	two-component sensor histidine kinase	17	225
COO91_01704	Signal transduction histidine kinase regulating C4-dicarboxylate transport system	16	63
COO91_07377	carotenoid cleavage dioxygenase	16	52

Table S2. Accession numbers for cyanobacterial proteins harboring *mys* gene clusters included in the phylogenetic analysis (Figures S2 and S3).

Cyanobacterial strains	Habitat	Genus	Dsp1	OrrA	MysA	MysB	MysC1	MysC2	MysC3	MysD	NRP
Anabaena azotica FACHB-119	Terrestrial	Nostocales	MBD2499337	MBD2505470	MBD2504765	MBD2504764	MBD2504763				MBD2504762
Anabaena sp. CCY 9910	Terrestrial	Nostocales	WP_414755268	WP_414753958	WP_414754320	WP_414754321	WP_414754322				WP_414754324
Anabaena subtropica FACHB-260	Terrestrial	Nostocales	MBD2345420	MBD2346850	MBD2346876	MBD2346875	MBD2346874				MBD2346873
Aphanothece sp. CMT-3BRIN-NPC111	terrestrial	Chroococcales	MBW4576210	MBW4577519	MBW4576373	MBW4576372		MBW4576371	MBW4576370	MBW4576369	
Calothrix parietina FACHB-288	freshwater	Nostocales	MBD2199632	MBD2196046	MBD2198281	MBD2198282	MBD2198283			MBD2198284	
Calothrix sp. FACHB-168	freshwater	Nostocales	MBD2206232	MBD2205980	MBD2202850	MBD2202849	MBD2202848			MBD2202847	
Chlorogloeopsis fritschii C42 A2020 084	freshwater	Nostocales	MBF2005099	MBF2005903	MBF2005730	MBF2005731	MBF2005732				MBF2005733
Chlorogloeopsis fritschii PCC 6912	Terrestrial	Nostocales	RUR80209	RUR73497	RUR75920	RUR75919	RUR75918				RUR75917
Chlorogloeopsis sp. ULAP01	Terrestrial	Nostocales	MDM9384596	MDM9379923	MDM9379850	MDM9379849	MDM9379848				MDM9379847
Chlorogloeopsis sp. ULAP02	Terrestrial	Nostocales	MFQ4146742	MFQ4144328	MFQ4146583	MFQ4146582	MFQ4146581				MFQ4146580
Coleofasciculus sp. FACHB-1120	freshwater	Coleofasciculales	MBD2741929	MBD2740198	MBD2744706	MBD2744705		MBD2744704	MBD2744703	MBD2744702	
Crinalium epipsammum PCC 9333	terrestrial	Gomontiellales	AFZ14518	AFZ14199	AFZ13246	AFZ13245		AFZ13244	AFZ13243	AFZ13242	
Cylindrospermum sp. NIES-4074	terrestrial	Nostocales	BAZ27915	BAZ29994	BAZ28361	BAZ28362	BAZ28363			BAZ28364	
Cylindrospermum stagnale PCC 7417	terrestrial	Nostocales	AFZ24617	AFZ25134	AFZ23628	AFZ23629		AFZ23631	AFZ23632	AFZ23630	
Dendronalium phyllosphericum CENA369	host	Nostocales	MBH8576053	MBH8574160	MBH8573799	MBH8573800	MBH8573801			MBH8573803	
Desmonostoc muscorum CCALA 125	terrestrial	Nostocales	MBX9252991	MBX9254536	MBX9256902	MBX9256901	MBX9256900			MBX9256899	
Floridaenema aerugineum BLCC-F46	freshwater	Aerosakkonematales	MFB2875284	MFB2880028	MFB2877955	MFB2877954	MFB2877953			MFB2877952	
Floridaenema flaviceps BLCC-F50	freshwater	Aerosakkonematales	MFB2895833	MFB2897959	MFB2895699	MFB2895698	MFB2895697			MFB2895696	
Komarekiella atlantica HA4396-MV6	terrestrial	Nostocales	MBW4685483	MBW4689424	MBW4686977	MBW4686978	MBW4686979			MBW4686980	
Mastigocladus laminosus UU774	freshwater	Nostocales	TFI53148	TFI54698	TFI54009	TFI54010	TFI54011				TFI54013
Mastigocoleus sp. MO 188.B34	marine	Nostocales	MDJ0693408	MDJ0694611	MDJ0693635	MDJ0693634	MDJ0693633				MDJ0693632
Mastigocoleus testarum BC008	marine	Nostocales	KST66950	KST70006	KST62693	KST62692	KST62691				KST62690
Microcoleus sp. herbarium12	terrestrial	Oscillatoriales	WP_333471823	WP_333471422	WP_333469025	WP_333469024	WP_333469023			WP_333469022	
Microcoleus sp. PH2017 12 PCY D A	terrestrial	Oscillatoriales	MCC3519428	MCC3476757	MCC3478271	MCC3478270	MCC3478269			MCC3478268	
Microcystis aeruginosa F13-15	freshwater	Chroococcales	NCS27930	NCS27072	NCS31291	NCS31292	NCS31293			NCS31294	
Microcystis aeruginosa PMC 728.11	freshwater	Chroococcales	MBE5229859	MBE5228432	MBE5228001	MBE5228000	MBE5227999			MBE5227998	
Microcystis sp. LE19-388.1G	freshwater	Chroococcales	MCZ8357354	MCZ8357170	MCZ8361082	MCZ8361081	MCZ8361080			MCZ8361079	
Nostoc calcicola FACHB-389	terrestrial	Nostocales	OKH42249	OKH33529	OKH32992	OKH32991	OKH32990			OKH32989	
Nostoc commune NIES-4072	terrestrial	Nostocales	GBG23322	GBG20657	GBG19206	GBG19205		GBG19203	GBG19202	GBG19204	
Nostoc desertorum CM1-VF14	terrestrial	Nostocales	MBW4427045	MBW4427965	MBW4424261	MBW4424260		MBW4424258	MBW4424257	MBW4424259	
Nostoc edaphicum CCNP1411	marine	Nostocales	QMS87340	QMS89537	QMS91208	QMS91209	QMS91210			QMS91211	
Nostoc favosum CHAB5714	terrestrial	Nostocales	MCC5600755	MCC5600066	MCC5597933	MCC5597934		MCC5597936	MCC5597937	MCC5597935	
Nostoc flagelliforme CCNUN1	terrestrial	Nostocales	AUB43086	AUB37308	AUB40031	AUB40032		AUB40034	AUB40035	AUB40033	
Nostoc linckia z1	terrestrial	Nostocales	PHJ63530	PHJ67243	PHJ57425	PHJ57424	PHJ57423			PHJ57422	
Nostoc minutum NIES-26	terrestrial	Nostocales	RCJ37919	RCJ21923	RCJ36489	RCJ36490	RCJ36491			RCJ36492	
Nostoc parmelioides FACHB-3921	Terrestrial	Nostocales	MBD2254393	MBD2253542	MBD2254355	MBD2254356	MBD2254357				MBD2254358
Nostoc punctiforme PCC 73102	host	Nostocales	ACC81961	ACC80154	ACC83905	ACC83904	ACC83903			ACC83902	
Nostoc sp. ATCC 43529	freshwater	Nostocales	RCJ15487	RCJ25477	RCJ25793	RCJ25794	RCJ25795			RCJ25796	
Nostoc sp. CCCryo 231-06	terrestrial	Nostocales	MCL6754114	MCL6749501	MCL6749583	MCL6749582		MCL6749580	MCL6749579	MCL6749581	
Nostoc sp. CCY0012	Terrestrial	Nostocales	WP_414545420	WP_414544155	WP_414543936	WP_414543935	WP_414543934				WP_414543933
Nostoc sp. CHAB 5834	terrestrial	Nostocales	MCC5610687	MCC5608680	MCC5608862	MCC5608863		MCC5608865	MCC5608866	MCC5608864	
Nostoc sp. ChiQUE01a	host	Nostocales	MDZ8240539	MDZ8241316	MDZ8239003	MDZ8239002	MDZ8239001			MDZ8239000	
Nostoc sp. CmiSLP01	host	Nostocales	MGF1978914	MDZ8166673	MDZ8163566	MDZ8163567	MDZ8163568			MDZ8163569	
Nostoc sp. CmiVER01	terrestrial	Nostocales	MDZ8121569	MDZ8124851	MDZ8125750	MDZ8125751		MDZ8125753	MDZ8125754	MDZ8125752	
Nostoc sp. DedQUE01	host	Nostocales	MFN6498647	MFN6499751	MFN6495520	MFN6495519	MFN6495518			MFN6495517	
Nostoc sp. DedSLP01	host	Nostocales	MFN6440985	MFN6435659	MFN6437128	MFN6437129	MFN6437130			MFN6437131	
Nostoc sp. FACHB-110	freshwater	Nostocales	MBD2439823	MBD2436625	MBD2440374	MBD2440375	MBD2440376			MBD2440377	
Nostoc sp. FACHB-152	freshwater	Nostocales	MBD2449721	MBD2450464	MBD2451670	MBD2451671		MBD2451673	MBD2451674	MBD2451672	
Nostoc sp. FACHB-857	freshwater	Nostocales	MBD2679174	MBD2677768	MBD2676679	MBD2676680	MBD2676681			MBD2676682	
Nostoc sp. FACHB-888	terrestrial	Nostocales	MBD2247853	MBD2242811	MBD2246838	MBD2246837		MBD2246835	MBD2246834	MBD2246836	
Nostoc sp. FACHB-892	Terrestrial	Nostocales	MBD2728914	MBD2728033	MBD2729429	MBD2729430		MBD2729432	MBD2729433	MBD2729431	
Nostoc sp. FACHB-973	freshwater	Nostocales	MBD2520306	MBD2518896	MBD2515221	MBD2515220	MBD2515219			MBD2515218	
Nostoc sp. GBBB01	freshwater	Nostocales	MBL1202667	MBL1203484	MBL1200040	MBL1200041	MBL1200042			MBL1200043	
Nostoc sp. HG1	terrestrial	Nostocales	MBC6430663	MBC6432888	MBC6430530	MBC6430529		MBC6430527	MBC6430526	MBC6430528	
Nostoc sp. KVJ20	host	Nostocales	ODH02315	ODG99321	ODH00099	ODH00100	ODH00101			ODH00102	
Nostoc sp. LLY	Terrestrial	Nostocales	MCG6137842	MCG6136367	MCG6137776	MCG6137777	MCG6137778				MCG6137779
Nostoc sp. MS1	freshwater	Nostocales	BCL38312	BCL38031	BCL38699	BCL38698	BCL38697				BCL38696
Nostoc sp. NIES-3756	Terrestrial	Nostocales	BAT55881	BAT54861	BAT53044	BAT53043	BAT53042				BAT53041
Nostoc sp. NIES-4103	freshwater	Nostocales	BAZ48701	BAZ51161	BAZ51203	BAZ51204	BAZ51205			BAZ51206	
Nostoc sp. NMS8	terrestrial	Nostocales	MBN3957359	MBN3961473	MBN3961043	MBN3961042		MBN3961040	MBN3961039	MBN3961041	
Nostoc sp. NOS(2021)	host	Nostocales	MBN3895717	MBN3896332	MBN3897427	MBN3897428		MBN3897430	MBN3897431	MBN3897429	
Nostoc sp. PCC 7524	freshwater	Nostocales	AFY47832	WP_041555503	AFY49165	AFY49164	AFY49163				AFY49162
Nostoc sp. 'Peltigera membranacea cyanobiont' 232	host	Nostocales	OYE06566	OYE03839	OYE01830	OYE01831		OYE01833	OYE01834	OYE01832	
Nostoc sp. 'Peltigera membranacea cyanobiont' N6	host	Nostocales	AVH62073	AVH64860	AVH64432	AVH64433		AVH64435	AVH64436	AVH64434	
Nostoc sp. S13	terrestrial	Nostocales	MDF5738632	MDF5734279	MDF5734608	MDF5734609		MDF5734611	MDF5734612	MDF5734610	
Nostoc sp. T09	host	Nostocales	OUL32867	OUL21206	OUL34647	OUL34648	OUL34649			OUL34650	
Nostoc sp. UHCC 0251	terrestrial	Nostocales	MEA5627473	MEA5623918	MEA5622765	MEA5622766		MEA5622768	MEA5622769	MEA5622767	
Nostoc sp. UHCC 0702	freshwater	Nostocales	QSJ15056	QSJ18384	QSJ18441	QSJ18442	QSJ18443			QSJ18444	
Nostoc sp. XA013	terrestrial	Nostocales	MCC5653597	MCC5650072	MCC5648324	MCC5648323		MCC5648321	MCC5648320	MCC5648322	
Nostoc sp. ZfuVER08	host	Nostocales	MDZ8016259	MDZ8011293	MDZ8013659	MDZ8013658	MDZ8013657			MDZ8013656	
Nostoc sphaeroides CCNUC1	freshwater	Nostocales	QFS51078	QFS46485	QFS49200	QFS49201	QFS49202			QFS49204	
Oscillatoria nigro-viridis PCC 7112	terrestrial	Oscillatoriales	AFZ06968	AFZ06036	AFZ08125	AFZ08126	AFZ08127			AFZ08128	
Pelatocladus maniniholoensis HA4357-MV3	Terrestrial	Nostocales	MBW4430471	MBW4430324	MBW4433483	MBW4433484	MBW4433485				MBW4433486
Roholtiella sp. LEGE 12411	freshwater	Nostocales	MBE9037491	MBE9035225	MBE9035861	MBE9035860	MBE9035859			MBE9035858	
Scytonematopsis contorta HA4267-MV1	freshwater	Nostocales	MBW4511762	MBW4505204	MBW4511447	MBW4511446	MBW4511445				MBW4511444
Trichormus variabilis ATCC 29413	freshwater	Nostocales	ABA25205	ABA21181	ABA23463	ABA23462	ABA23461				ABA23460
Tychonema sp. LEGE 07203	terrestrial	Oscillatoriales	MBE9094941	MBE9097295	MBE9095633	MBE9095634	MBE9095635			MBE9095636	
Xenococcaceae cyanobacterium MO 207.B15	marine	Pleurocapsales	MDJ0533203	MDJ0534082	MDJ0535125	MDJ0535124	MDJ0535123				MDJ0535121

Table S3. Primers used in this study.

Primer name	5'—3'
RT-mysA-For	GGTTTGCTTGCGCTTCTTAC
RT-mysA-Rev	CCAACTCTGCCATCCCATTA
RT-mysB-For	AATGTTCACAGGGTATTCAGCC
RT-mysB-Rev	CCGTCGGGAGCCAGTAAGT
RT-mysD-For	CGATCGTTCGGTTTATCTGTC
RT-mysD-Rev	TCCAGGGGTTCTCCTCACTAA
RT-mysC2-For	ACCGTCGTACTTGCATCGC
RT-mysC2-Rev	AAGGCAGCCACAGAGTTAGA
RT-mysC3-For	CGGGCACTATTCGACTTGC
RT-mysC3-Rev	CGGGTTTGATGACTACTGGAGG
RT-dsp1-For	ACCGTCGTACTTGCATCGC
RT-dsp1-Rev	AAGGCAGCCACAGAGTTAGA
RT-orrA-For	CCGTAAATTCAAACGCCACC
RT-orrA-Rev	GCATCTTCCGCAATACCACA
RT-susA-For	TCATCCGCGACCCCAAAAAT
RT-susA-Rev	TGCTGCTGTGATTGAATGCG
RT-susB-For	ACCTCCACCCGTTTTACGAAA
RT-susB-Rev	CTCAACAACAGCCGAATTCCA
RT-treZ-For	CTAACTACTTGGGTGACGGAGAGT
RT-treZ-Rev	GATAAAGCGTGCCTGGATATACAT
RT-16s-For	AGGTGGCTGTGTAAGTCTGCTGT
RT-16s-Rev	TTCGCCACTGGTGTTCTTCCTG
Y2H-orrA-Hik-AD-For	CGCCATATGATTAGCATTATTTTAATTGA
Y2H-orrA-Hik-AD-Rev	CCGCTCGAGTAGCGCCCTAACAGCAGCTT
Y2H-dsp1-BD-For	CGCCATATGGAACGTAACCGTATGGCACG
Y2H-dsp1-BD-Rev	GCTGCAGTCCTTGATTGACTATGACAG
Y1H-susB-For	ATCGGTACCACACTGCGTGTAACTAACACTG
Y1H-susB-Rev	ATCGTCGACGATGAGGAATCCCAGAACTTTA
PD-dsp1-GST-For	CGGAATTCGAACGTAACCGTATGGCACG
PD-dsp1-GST-Rev	CCGCTCGAGTCCTTGATTGACTATGACAG
PD-orrA-His-For	CGGAATTCATTAGCATTATTTTAATTGA
PD-orrA-His-Rev	CCGCTCGAGTAGCGCCCTAACAGCAGCTT
CRISPR-gd_mysA-F	AGATATATTCAAAACAGAGAAATAGC
CRISPR-gd_mysA-R	AGACGCTATTTCTCTGTTTTGAATAT
CRISPR-mysA-F1	TCGATATCTAGATCTCATGGATCGCTCAAGAGCCAGTCTAGTAGAGA
CRISPR-mysA-R1	TATAGCAGTTCAAAGACTTCAGAAGTGTGTTGACCTTATTCCAATTAA
CRISPR-mysA-F2	TTAATTGGAATAAGGTCAACACACTTCTGAAGTCTTTGAACTGCTATA
CRISPR-mysA-R2	CGTTGTTGCCATTGCGGATCGAATACCCTGTGAACATTCCTACT
CRISPR-gd_mysC3-F	AGATTCAAAAGTCTGCCCATTAGTGA
CRISPR-gd_mysC3-R	AGACTCACTAATGGGCAGACTTTTGA
CRISPR-mysC3-F1	TCGATATCTAGATCTCATGGATCCCAGTGTTATCAGGCTATTGC
CRISPR-mysC3-R1	GAAGTCAAAGGAAAGTTGCCCTACAAAAATATGCTTTCTCATACT

CRISPR-mysC3-F2	AGTATGAGAAAGCATATTTTTGTAGGGCAACTTTCCTTTGACTTC
CRISPR-mysC3-R2	CGTTGTTGCCATTGCGGATCGTGTTTGGTTCTCCTCTACCA
CRISPR-gd_dsp1-F	AGATACGAGCAGGGCAATGTGACTTA
CRISPR-gd_dsp1-R	AGACTAAGTCACATTGCCCTGCTCGT
CRISPR-dsp1-F1	GCAGAAATTCGATATCTAGATCAAACAAGAAACAGCACTAACCTGG
CRISPR-dsp1-R1	AAAGCCTTGTTTGATTTTCAAGATACAACACGCC
CRISPR-dsp1-F2	GAAAATCAAACAAGGCTTTGGAGTAGGTAGCGT
CRISPR-dsp1-R2	CGCAACGTTGTTGCCATTGCTGAAAGTTAACCGAAAGCACAGCC
CRISPR-gd_orrA-F	AGATACTCAACGGGAACTAGAAATCC
CRISPR-gd_orrA-R	AGACGGATTTCTAGTTCCCGTTGAGT
CRISPR-orrA-F1	GCAGAAATTCGATATCTAGATCAAGTCAGACCAACAAGCTTCCCCC
CRISPR-orrA-R1	TAGTTTATTTTGTAATAAGAATCAGCACCCGCCG
CRISPR-orrA-F2	TTCTTATTACAAAATAAACTATGTGCTGATGACCG
CRISPR-orrA-R2	CGCAACGTTGTTGCCATTGCGAGATATTGTCCCACTGTTGTTTG

The RT primers were used for quantitative reverse transcription polymerase chain reaction (qRT-PCR). Y2H and Y1H primers were used for the yeast two-hybrid and yeast one-hybrid assays, respectively. The PD primers were applied in pull-down assays. CRISPR primers were employed in the generation of *N. flagelliforme* CCNUN1 mutants.

Table S4. Strains used in this study.

Strains	Antibiotics	For study
Nostoc flagelliforme CCNUN1	None	Genomic DNA as a template for PCR, used as a control
Δ*mysA* (*N. flagelliforme* CCNUN1)	None	Phenotype analysis of water-deficit tolerance
Δ*mysC3* (*N. flagelliforme* CCNUN1)	None	Phenotype analysis of water-deficit tolerance
Δ*orrA* (*N. flagelliforme* CCNUN1)	None	Phenotype analysis of water-deficit tolerance
Δ*dsp1* (*N. flagelliforme* CCNUN1)	None	Phenotype analysis of water-deficit tolerance
